# Strategically Constructing Alkali‐Metal Interfacial Bridges to Boost Photocatalytic CO_2_ Methanation on Supported Ni─Ru Bimetallic Catalysts

**DOI:** 10.1002/advs.202509454

**Published:** 2025-08-04

**Authors:** Xiaolei Guo, Yuqi Wu, Shengrong Zhou, Yuhang Shao, Yasuo Izumi, Jinlu He, Hongwei Zhang

**Affiliations:** ^1^ College of Chemistry and Chemical Engineering Inner Mongolia University Hohhot 010021 P. R. China; ^2^ Key Laboratory of Development and Application of Rural Renewable Energy Biogas Institute of Ministry of Agriculture and Rural Affairs Chengdu 610041 P. R. China; ^3^ Department of Chemistry Graduate School of Science Chiba University Yayoi 1–33 Inage‐ku Chiba 263–8522 Japan

**Keywords:** alkali‐metal interfacial bridges, bimetallic sites, CO_2_ methanation, metal‐support interactions, photocatalysis

## Abstract

Efficient photocatalytic conversion of CO_2_ into CH_4_ is crucial yet challenging due to the complex multi‐electron transfer processes and sluggish intermediate transformation. Herein, an innovative strategy is introduced to dramatically enhance photocatalytic CO_2_ methanation by constructing interfacial alkali‐metal bridges (Na_inter_) between Ni and Ru nanoparticles over ZrO_2_ surface. By selectively introducing and subsequently removing excessive surface Na species, stable interfacial Na species are retained, forming a distinctive Ni^0^─Ni^δ+^─Na_inter_─O─Ru electronic bridge. Comprehensive structural and electronic characterizations (XRD, TEM, XAFS, XPS, DRIFTS) demonstrate that the interfacial Na bridge significantly improves electronic communication between Ni and Ru, enhances charge separation efficiency, optimizes CO_2_ adsorption, and lowers activation barriers for key intermediates. As a result, the optimized catalyst (0.2Na─Ni─Ru/ZrO_2_) achieves an exceptionally high CH_4_ production rate of 1882.7 µmol·g^−1^·h^−1^, ≈15‐fold that of the Na‐free catalyst, with excellent stability and durability. DFT calculations reveal that the Na_inter_ site effectively stabilizes reactive intermediates, greatly accelerating formate to CO conversion and reshaping the reaction pathway. This work highlights alkali‐metal‐mediated interfacial engineering as a versatile approach to enhance the synergy in multi‐component catalysts, opening a new avenue for advanced photocatalytic CO_2_ reduction.

## Graphical abstract

Strategically anchoring Na at the Ni─Ru/ZrO_2_ interface creates a robust Ni^0^─Ni^δ+^─Na─O─Ru electronic bridge, significantly enhancing electron transfer and inter‐metal synergy. This interface accelerates photocatalytic CO_2_ methanation via stabilized *COOH intermediates and efficient H_2_ activation, boosting CH_4_ yield.

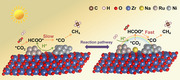

## 1 Introduction

The escalating global environmental crisis, driven predominantly by excessive fossil fuel consumption and resulting CO_2_ emissions, necessitates innovative approaches for sustainable energy generation and carbon mitigation. Photocatalytic CO_2_ reduction, utilizing renewable solar energy to convert CO_2_ into methane (CH_4_), presents a compelling strategy for addressing these pressing challenges. CH_4_ as a versatile carbon‐neutral fuel, is readily integrated into existing natural gas infrastructures, enabling efficient energy storage and distribution while serving as a precursor for essential chemicals and fuels.^[^ ^1^ ^–^ ^4^ ^]^

The photocatalytic reduction of CO_2_ to CH_4_ is inherently complex, involving multiple electron–proton transfer processes and various intermediate species adsorbed on catalyst surfaces. Single‐metal photocatalysts are typically limited by isolated active sites, insufficient charge‐carrier separation, and significant kinetic barriers associated with intermediate transformations.^[^ ^2^, ^5^ ^]^ Bimetallic photocatalysts have emerged as a promising alternative due to their potential to leverage cooperative interactions between two distinct metallic components, enhancing the electronic structure and facilitating efficient intermediate adsorption and conversion.^[^ ^5^, ^6^, ^7^, ^8^ ^]^ Specifically, Ni─Ru bimetallic catalysts have attracted considerable attention owing to Ru's superior capability for hydrogen activation and spillover effects, maintaining Ni in its active metallic state. The addition of Ru as a promoter is found to significantly increase the amount of medium‐strength basic sites, which is recognized as an important factor enhancing CO_2_ adsorption and activation, thus promoting CO_2_ activation and subsequent hydrogenation reactions. Nevertheless, precise control of the interfacial interactions between the two metallic phases to achieve optimal synergy remains an unresolved challenge, hindering the full exploitation of their catalytic potential.^[^ ^9^ ^]^

Recent advances highlight the promising role of alkali metals, such as K‐modified Ni─Zn bimetallic catalysts can form a K‐Ni_3_Zn_1_C_0.7_ active phase, significantly enhancing CO_2_ adsorption and thereby promoting the formation of C_2_^+^ alcohols upon CO_2_ hydrogenation.^[^ ^10^ ^]^ Additionally, introducing alkali‐metal Na into Rh_1_/ZrO_2_ catalysts can regulate the synergy between metal single atoms and the support in terms of both electronic structure and geometric configuration, thereby altering product selectivity during CO_2_ hydrogenation.^[^ ^11^ ^]^ Moreover, it has been reported that Au single atoms can undergo redispersion at the interface of a Na^+^‐modified TiO_2_ support, demonstrating that alkali metal incorporation significantly alters metal–support interactions (MSI) and effectively anchors active metal species.^[^ ^12^ ^]^ Additionally, alkali metals enrich the charge density at the metal–support interface region and simultaneously enhance the adsorption strengths of CO_2_ and CO, thereby promoting the CO_2_ methanation reaction.^[^ ^13^, ^14^ ^]^ Inspired by these insights, this work explores an innovative approach involving the deliberate construction of an interfacial alkali‐metal bridge to enhance the cooperative catalytic behavior of Ni─Ru bimetallic catalysts for photocatalytic CO_2_ methanation.

In this study, Na is strategically introduced during the catalyst synthesis to preferentially occupy surface sites on the ZrO_2_ support, followed by deposition of Ni and Ru nanoparticles. After controlled reduction treatments and washing to remove loosely bound surface Na species (Na_sur_), a stable interfacial Na species (Na_inter_) is retained. Comprehensive characterization techniques reveal that Na_inter_ effectively bridges Ni and Ru nanoparticles, forming a unique electronic pathway denoted as Ni^0^─Ni^δ+^─Na_inter_─O─Ru. This interfacial configuration significantly modifies the electronic environment at the catalyst interface, promoting enhanced electron transfer and inter‐metallic synergy. Consequently, the optimized catalyst, 0.2Na─Ni─Ru/ZrO_2_, exhibits a remarkable photocatalytic CH_4_ generation rate of 1882.7 µmol·g^−1^·h^−1^, representing approximately a 15‐fold increase compared to the control catalyst without the alkali‐metal interface. The developed interfacial engineering strategy provides a new paradigm for precise tuning metal–metal and MSI, offering valuable guidance for future catalyst design in photocatalytic CO_2_ conversion.

## 2 Results and Discussion

### 2.1 Catalyst Synthesis and Structural Analysis

**Figure 1:**
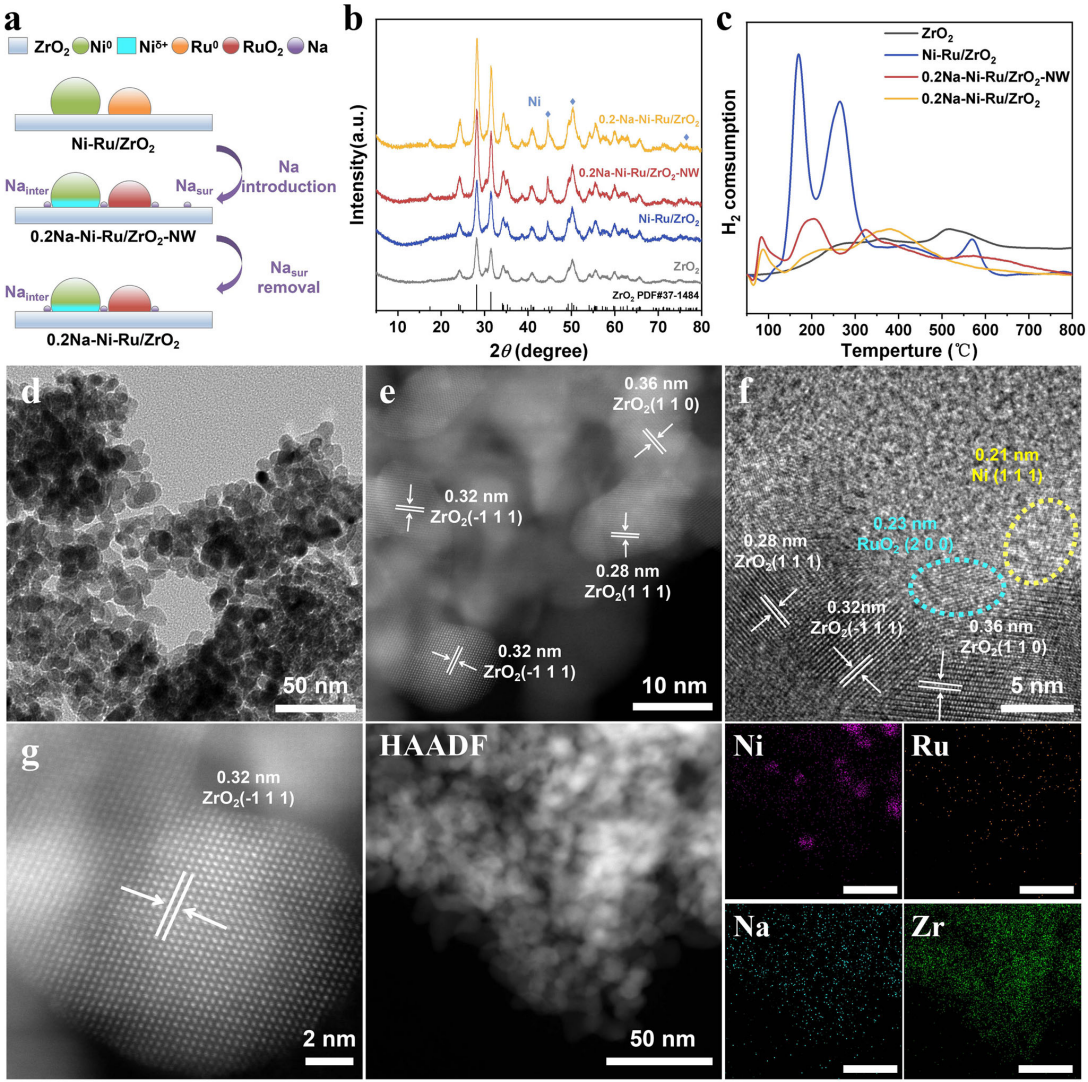
a) Schematic illustration of the structural design strategy for Ni‐Ru/ZrO_2_ catalysts with interfacial alkali‐metal bridges; b) XRD patterns; c) H_2_‐TPR profiles of ZrO_2_, Ni‐Ru/ZrO_2_, 0.2Na‐Ni‐Ru/ZrO_2_‐NW, and 0.2Na‐Ni‐Ru/ZrO_2_ catalysts. d,f) TEM images, e,g) aberration corrected STEM images and corresponding HAADF‐STEM image with EDX elemental mappings for Ni, Ru, Na, and Zr in the optimized 0.2Na‐Ni‐Ru/ZrO_2_ catalyst.

A series of Ni─Ru/ZrO_2_ catalysts (10 wt.% Ni, 1.5 wt.% Ru) was prepared by impregnation, with NaNO_3_ introduced in the initial step to construct an interfacial alkali metal bridge (**Figure** **1**a). The Na species pre‐anchored on the ZrO_2_ support surface during synthesis serve as nucleation sites for Ni─Ru deposition, leveraging the known ability of alkali promoters to anchor metal atoms and modulate MSI. After hydrogen reduction (450 °C in H_2_/Ar), following our previously reported, the unwashed sample is denoted xNa─Ni─Ru/ZrO_2_‐NW (“not washed”), where x = molar ratio of Na/Ni. Subsequent thorough water‐washing removes excess surface Na (Na_sur_) while retaining strongly bound interfacial Na (Na_inter_) at the metal–support interface. The finally washed catalysts are labeled xNa─Ni─Ru/ZrO_2_ (x = 0.05‐1.0). X‐ray diffraction (XRD) patterns (Figure S1, Supporting Information) show no distinct Ru peaks due to low Ru loading, while reflections for ZrO_2_ and Ni are evident. Notably, the Ni(111) and (200) diffraction peaks intensify upon Na introduction (0.2Na─Ni─Ru/ZrO_2_ vs Ni─Ru/ZrO_2_), indicating larger Ni crystallites. The unwashed 0.2Na─Ni─Ru/ZrO_2_‐NW sample exhibits similarly enhanced Ni peaks, confirming that pre‐loading Na species promotes Ni deposition via an anchoring effect. These results suggest that an interfacial Na bridge can be successfully established between Ni and Ru on ZrO_2_ during synthesis.

Hydrogen temperature‐programmed reduction (H_2_‐TPR) profiles (Figure 1c; Figure S2, Supporting Information) provide insight into the catalyst reduction behaviors. Bare ZrO_2_ shows a broad H_2_ consumption peak at 450–600 °C, attributed to the partial reduction of surface lattice oxygen. Upon loading Ni─Ru, new reduction features appear at much lower temperatures: a peak ≈170 °C corresponding to the reduction of RuO_2_ and small NiO species, and another ≈ 265 °C for larger NiO particles.^[^ ^8^, ^15^, ^16^ ^]^ This indicates that the as‐prepared Ni─Ru/ZrO_2_ contains Ni mostly as NiO alongside RuO_2_, rather than a fully alloyed Ni─Ru phase. Introducing Na_inter_ markedly alters the TPR profile: in 0.2Na─Ni─Ru/ZrO_2_, the sharp NiO/RuO_2_ peaks are suppressed and instead two broad reduction bands appear at 140–280 °C and 285–560 °C. The greatly decreased H_2_ consumption (relative to Ni─Ru/ZrO_2_) suggests fewer reducible surface oxides rather than an inability to split H_2_ (the surface predominantly remains in a metallic state), consistent with Na anchoring inducing growth of larger Ni─Ru nanoparticles, lowering their surface‐to‐bulk ratio and thus reducing the amount of surface oxide that forms. Moreover, the shift of reduction features to higher temperature ranges indicates a stronger metal–support interaction in the Na‐bridged catalyst.^[^ ^17^ ^]^ It is noteworthy that both 0.2Na─Ni─Ru/ZrO_2_‐NW and the washed 0.2Na sample exhibit a minor H_2_ uptake at ≈95–120 °C, attributable to H_2_ adsorption on metallic Ni/Ru surfaces. This low‐temperature uptake implies that the introduction of Na_inter_ improves the resistance of Ni/Ru to oxidation (more metallic sites remain available), in line with the Ni K‐edge XANES results (discussed below). Thus, the interfacial Na bridge not only anchors Ni─Ru particles but also enhances their interaction with ZrO_2_ and preserves metallic species during oxidative handling.

Transmission electron microscopy (TEM) and related analyses revealed the morphological differences upon Na incorporation. As shown in Figure 1d–f, the ZrO_2_ support consists of nanoparticles ≈10–20 nm in size. Both Ni and Ru atoms form nanoparticles (≈5 nm) well‐dispersed on the support in 0.2Na─Ni─Ru/ZrO_2_, slightly larger on average than those in Ni─Ru/ZrO_2_ (which are primarily <5 nm, Figure S3, Supporting Information). High‐resolution TEM images identify lattice fringes with spacings of 0.197 and 0.207 nm, corresponding to Ni(200) and Ni(111) planes, and 0.234/0.240 nm for Ru(100). Larger lattice spacings of 0.267–0.367 nm are ascribed to various ZrO_2_ crystal planes.^[^ ^18^, ^19^, ^20^ ^]^ These observations confirm that metallic Ni and Ru nanoparticles are present and crystalized oriented on ZrO_2_. Consistent with the XRD/TPR inference, the Na‐introduced sample shows slightly enlarged Ni─Ru particles, reflecting the Na‐induced aggregation/anchoring effect. High‐angle annular dark‐field STEM (HAADF‐STEM) coupled with energy‐dispersive X‐ray (EDX) mapping further confirms the presence and location of Na (Figure 1g–k). In 0.2Na─Ni─Ru/ZrO_2_, Na signals overlap with Ni, Ru, and Zr, indicating that Na is situated at the Ni/Ru─ZrO_2_ interface. The unwashed sample (0.2Na─Ni─Ru/ZrO_2_‐NW) shows a considerably stronger Na signal in HAADF/EDX (Figure S4, Supporting Information), which decreases after washing, evidencing effective removal of excess surface Na. X‐ray photoelectron spectroscopy (XPS) analysis of Na 1s corroborates this: 0.2Na─Ni─Ru/ZrO_2_‐NW displays a pronounced Na 1s peak at 1072.4 eV that extensively decreases upon washing (Figure S5, Supporting Information). Inductively coupled plasma (ICP) results quantify the Na content dropping from 0.795 wt.% in 0.2Na─Ni─Ru/ZrO_2_‐NW to only 0.045 wt.% after washing. Collectively, these results verify that the post‐synthesis washing step removes labile Na_sur_ species while retaining a minute amount of strongly bound Na_inter_ at the Ni─Ru/ZrO_2_ interface. This interfacial Na is pivotal for constructing the intended “Ni─Na─Ru” bridges surface.

**Figure 2:**
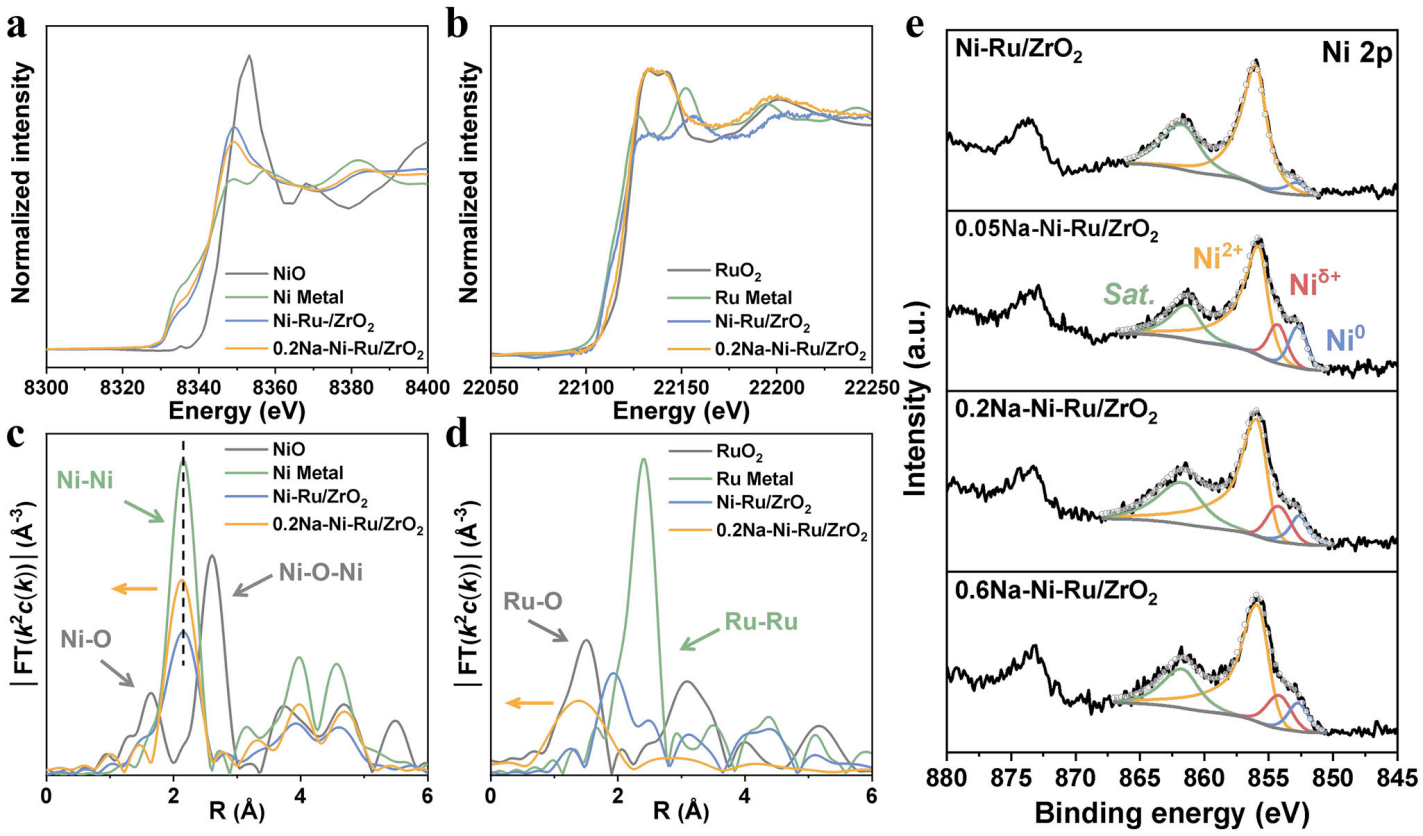
Electronic structure characterization of catalysts. a,b) Ni K‐edge and Ru K‐edge X‐ray absorption near‐edge structure (XANES); c,d) corresponding extended X‐ray absorption fine structure (EXAFS) spectra of Ni‐Ru/ZrO_2_ and 0.2Na‐Ni‐Ru/ZrO_2_ catalysts; e) High‐resolution Ni 2p X‐ray photoelectron spectra (XPS) showing the evolution of oxidation states upon varying Na content.

To understand the electronic effects of the Na_bridge_, we performed X‐ray absorption fine spectroscopy (XAFS) at the Ni and Ru K‐edges, as well as XPS and electron paramagnetic resonance (EPR) on the series of catalysts. The Ni K‐edge X‐ray absorption near‐edge structure (XANES) spectra (**Figure** **2**a) show that Ni in both Ni─Ru/ZrO_2_ and 0.2Na─Ni─Ru/ZrO_2_ has an absorption edge between that of Ni^0^ (metallic Ni foil) and Ni^2+^ (NiO), indicating a mixed valence state. However, the white‐line intensity for 0.2Na─Ni─Ru/ZrO_2_ is lower and its edge position shifts closer to Ni^0^, suggesting that Ni is in a more reduced state compared to Ni─Ru/ZrO_2_.^[^ ^10^ ^]^ This implies Na_inter_ confers greater resistance to Ni oxidation (more Ni° character), consistent with the TPR observation of retained metallic adsorption sites. The Ni K‐edge extended XAFS (EXAFS) (Figure 2c) further reveals a prominent Ni─Ni coordination peak at ≈2.15 Å in both samples, confirming that Ni exists as metallic Ni clusters. In 0.2Na─Ni─Ru/ZrO_2_, the Ni─Ni peak amplitude is increased, consistent with larger Ni nanoparticles (greater coordination number) as inferred from TEM. Interestingly, a slight shift in the Ni─Ni peak position toward shorter bond side is observed for 0.2Na─Ni─Ru/ZrO_2_, suggesting an interaction between Ni and the interfacial Na (altering Ni's local environment).^[^ ^21^ ^]^

At the Ru K‐edge (Figure 2b), XANES reveals that Ru in both catalysts is also in an intermediate oxidation state between Ru^0^ and RuO_2_. In contrast to Ni, the 0.2Na─Ni─Ru/ZrO_2_ sample shows a white‐line intensity slightly closer to RuO_2_, indicating Ru is more oxidized (electron‐deficient) than in Ni─Ru/ZrO_2_. This observation aligns with the notion that Ni becomes more reduced at the expense of Ru becoming slightly oxidized when Na is introduced, hinting at electron transfer from Ru toward Ni via Na. The Ru K‐edge EXAFS (Figure 2d) provides striking evidence of altered Ru coordination. Ni─Ru/ZrO_2_ exhibits two notable features: one peak at a distance intermediate between Ru─Ru (metallic, ≈2.4 Å) and Ru─O (≈1.5 Å), and a second peak at ≈2.4 Å corresponding to Ru─Ru bonds. This suggests that in Ni─Ru/ZrO_2_, Ru exists in dual forms – as Ru metal (or Ni─Ru alloy) clusters and as oxidized Ru species interacting with oxygen (e.g., RuO_2_ domains or Ru at the metal–support interface). In stark contrast, 0.2Na─Ni─Ru/ZrO_2_ shows only a Ru─O contribution in the EXAFS, with the Ru─Ru metallic signal essentially absent.^[^ ^22^, ^23^, ^24^ ^]^ Interestingly, the expected Ru─(O)─Ru peak does not appear at the usual ≈3.1 Å but instead reaches a maximum near ≈2.9 Å. This shift is best interpreted as a convolution of Ru─(O)─Ru (≈3.1 Å) and Ru─(O)─Ni (≈2.6 Å) contributions. Combined with the slight contraction in the Ni─Ni distance of Ni^0^ induced by Na (discussed above), these results point to a coordination environment in which Ni and Ru are linked through an interfacial Na─O bond, giving rise to a Ni^0^─Na_inter_─RuO_2_ bridge. Moreover, HR–TEM reveals that the Ru oxide domains are very small (<5 nm), giving them a high surface to volume ratio. Consequently, the corresponding Ru─(O)─Ru (or Ru─(O)─Ni) peak appears with lower intensity than would be expected for bulk RuO_2_. These XAFS results clearly demonstrate that the presence of interfacial Na alters the electronic states: Ni is stabilized in a more reduced state, while Ru tends toward an oxidized state bound to oxygen, consistent with a Ni^0^─Ni^δ+^─Na_inter_─RuO_2_ bridging. In effect, Na_inter_ functions as an electron bridge between Ni and Ru, promoting electronic coupling and charge redistribution among the two metals and the support.

XPS measurements further elucidate these electronic modifications on the catalyst surface (Figure 2e; Figure S6, Supporting Information). No obvious Na 1s signal is detected on the XPS survey of the washed Na‐promoted samples (0.05Na, 0.2Na, 0.6Na), confirming the absence of Na_sur_after washing. The Ni 2p_3/2_ spectra (Figure 2e) show that in Ni─Ru/ZrO_2_, Ni exists mostly as Ni^2+^ (binding energy BE ≈ 855.9 eV) with a minor metallic Ni^0^ peak at ≈852.7 eV (Table S2, Supporting Information), due to surface oxidation when exposed to air.^[^ ^12^ ^]^ Importantly, upon Na promotion, a new Ni^δ+^ feature emerges at ≈852.2 eV (between Ni^0^ and Ni^2+^) for all Na‐containing samples. This indicates that Na_inter_ induces the formation of partially positively charged Ni species (0< δ <2). We infer that Ni atoms in close proximity to the Na bridge become electron‐deficient (Ni^δ+^) due to electron transfer toward the electronegative Na─O─Ru interface. In essence, a gradient of Ni oxidation states is established (Ni° core with Ni^δ+^ at the interface), constructing an Ni^0^─Ni^δ+^─Na_inter_ conduit for electron flow. This likely originates from Ni nanoparticles nucleating at Na‐rich sites, creating transitional valence Ni at the boundary (Figure 2e).

In parallel, the Zr 3d peaks shift to lower BE with increasing Na content (Figure S6c, Supporting Information), signifying that the support is more reduced in the presence of Na_inter_. We attribute this to enhanced hydrogen spillover or electron donation to the support facilitated by Na (as also evidenced by higher oxygen vacancy (V_o_) levels, see below). The O 1s spectra (Figure S6d, Supporting Information) can be deconvoluted into lattice oxygen (O_lat_ at ≈529.5 eV) and surface defect oxygen (O_def_ at ≈530.4–531.4 eV, including ─OH and V_o_).^[^ ^25^, ^26^, ^27^ ^]^ The proportion of O_def_ increases from 62.7% on Ni─Ru/ZrO_2_ to 66.5% on 0.2Na─Ni─Ru/ZrO_2_, indicating that Na introduction generates more oxygen vacancies or defect sites on the surface (Table S3, Supporting Information). Electron paramagnetic resonance (EPR, Figure S7, Supporting Information) further supports this, showing that while loading Ni─Ru already raises the concentration of V_o_ relative to bare ZrO_2_, the inclusion of Na_inter_ leads to an even stronger O_v_ signal.^[^ ^28^, ^29^ ^]^ These defects are known to promote electron transfer processes. All these evidence demonstrate that the Na bridge renders the catalyst surface more electron‐rich and defect‐rich, strengthening electronic interactions both between Ni and Ru and between the bimetallic particles and the support. Such electronic modulation is expected to favor the adsorption/activation of reactants and facilitate the CO_2_ methanation reaction.^[^ ^25^ ^]^

### 2.2 Photocatalytic Performance and Structure–Activity Effect

**Figure 3:**
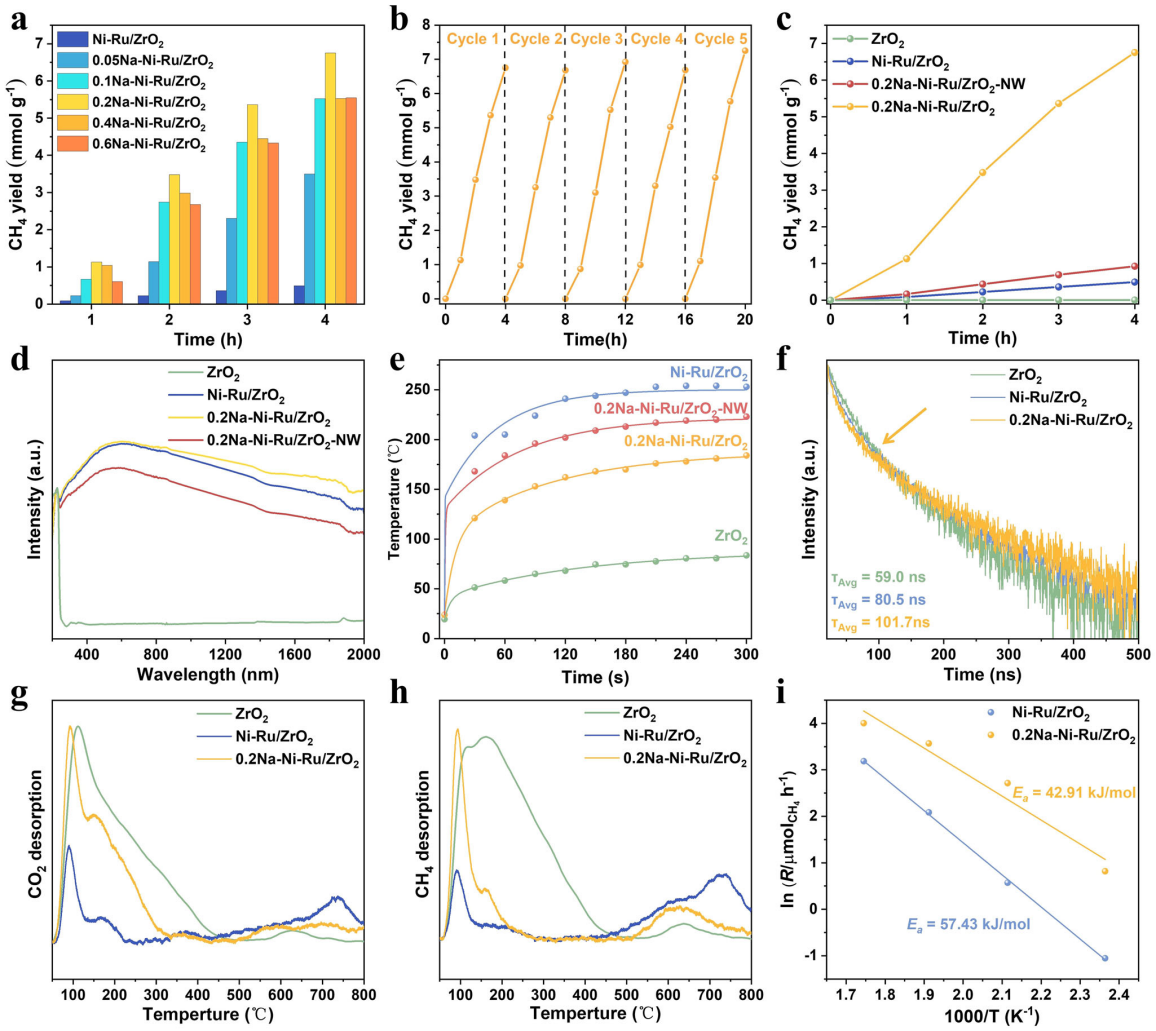
Photocatalytic CO_2_ methanation performance: a) CH_4_ evolution rates of xNa‐Ni‐Ru/ZrO_2_ catalysts (x = n(Na/Ni), 0.05‐0.6); b) Cycling stability test of the optimized 0.2Na‐Ni‐Ru/ZrO_2_ catalyst over 20 h; c) Comparison of Photocatalytic CO_2_ methanation rate among various catalysts; d) UV–vis diffuse reflectance spectra; e) Infrared thermal images under reaction conditions; f) TRPL decay curves; g,h) CO_2_ and CH_4_ TPD profiles; i) Arrhenius plots derived from CH_4_ production rates over Ni‐Ru/ZrO_2_ and 0.2Na‐Ni‐Ru/ZrO_2_.

We evaluated the photocatalytic CO_2_ methanation activity of the xNa─Ni─Ru/ZrO_2_ series under UV–vis illumination (1.5 W cm^−2^) in a closed‐circulation glass reactor system with mixture gas (CO_2_:H_2_:Ar = 1:9:10). Pristine ZrO_2_ shows negligible CH_4_ production, while Ni─Ru/ZrO_2_ exhibits measurable activity. Introducing Na dramatically boosts the performance (**Figure** **3**a). An optimal Na content exists: 0.2Na─Ni─Ru/ZrO_2_ achieves the highest CH_4_ yield, with a rate of 1882.7 µmol·g^−1^·h^−1^ ≈15 times greater than that of Ni─Ru/ZrO_2_ without Na, the turnover number (TON) of the 0.2Na─Ni─Ru/ZrO_2_ catalyst was approximately 4.3. Further increasing Na loading beyond a Na/Ni ratio of 0.4 shows no additional benefit (Figure S8, Supporting Information) and even slightly decrease the activity, likely because the surface sites for Na incorporation become saturated at ≈0.04 wt.% Na (Table S1, Supporting Information) and excess Na could form inoperative phases. The 0.2Na─Ni─Ru/ZrO_2_ catalyst also exhibits excellent stability, maintaining consistent CH_4_ production over five consecutive 4 h cycles (20 h total) with no deactivation (Figure 3b). The XRD patterns and TEM images of the 0.2Na─Ni─Ru/ZrO_2_ catalyst after five reaction cycles closely resemble those of the fresh catalyst, indicating excellent structural stability (Figure S9, Supporting Information). This stability under prolonged reaction suggests that the Na_inter_ species was robust and not leached or deactivated during photocatalysis. Similarly, the K‐doped catalyst also enhanced CH_4_ production compared to the undoped Ni─Ru/ZrO_2_ catalyst, confirming the general applicability of our alkali‐metal bridging strategy. Nevertheless, Na exhibited a notably more significant promotional effect under the present experimental conditions (Figure S10, Supporting Information).

To discern the roles of surface versus interfacial Na, we compared the unwashed and washed catalysts. The 0.2Na─Ni─Ru/ZrO_2_‐NW (with Na_sur_ present) shows a apparently lower CH_4_ rate than its washed counterpart (Figure 3c). The Na_sur_‐rich sample's activity is even inferior to Ni─Ru/ZrO_2_, indicating that excessive Na on the surface poisons active sites or blocks light absorption. In contrast, the washed sample, containing only Na_inter_, retains the high activity, confirming that interfacial Na is the key promoter while residual surface Na is detrimental. Thus, the water‐washing step to remove Na_sur_ is critical for performance enhancement.

The presence of Na_sur_ versus Na_inter_ also modulates the catalysts’ optical absorption and photothermal behavior. UV–vis diffuse‐reflectance spectra (Figure 3d) reveal that loading Ni and Ru markedly strengthens light harvesting across the full UV–visible–NIR region, extending effective absorption over 200–2000 nm. Despite this broader response, all photocatalysts retain an absorption edge at 248 nm (bandgap ≈ 5.0 eV), indicating that introducing metals enhances photon‐capture efficiency without narrowing the intrinsic bandgap. The sample containing excess surface sodium (0.2 Na─Ni─Ru/ZrO_2_‐NW, rich in Na_sur_) shows a slightly weaker absorption profile than its washed counterpart (0.2 Na─Ni─Ru/ZrO_2_). We ascribe this attenuation to surplus Na_sur_ partially masking the ZrO_2_ surface, thereby limiting effective photon absorption by the semiconductor and reducing photogenerated charge density. In contrast, the catalyst bearing only interfacial sodium (Na_inter_) displays absorption comparable to—or even higher than that of Ni─Ru/ZrO_2_. This enhancement is likely due to electronic modulation by Na at the Ni/Ru─ZrO_2_ interface (e.g., the creation of localized states), which modestly broadens the light‐absorption (Figure S11, Supporting Information).^[^ ^28^ ^]^

Consistent with the absorption behavior, infrared thermal imaging reveals pronounced photothermal heating of the catalysts under illumination (Figure 3e; Figure S12, Supporting Information). The Ni─Ru/ZrO_2_ sample heats up to the highest steady‐state surface temperature (≈253 °C) due to efficient light absorption and plasmonic/thermal effects of Ni─Ru, far above the 83.6 °C reached by bare ZrO_2_ (which has minimal absorption). Interestingly, the 0.2Na─Ni─Ru/ZrO_2_‐NW sample reaches a slightly lower temperature (≈223 °C), consistent with its reduced light absorption (Na_sur_‐induced light blocking). The 0.2Na─Ni─Ru/ZrO_2_ (≈184 °C)without Na_sur_ likely attains a similar high temperature to Ni─Ru within experimental variance, indicating that preserving only Na_inter_ does not diminish the photothermal effect. In summary, removing surface Na restores strong light absorption and heating, which can enhance reaction rates by providing additional thermal energy under reaction conditions. Moreover, in a control experiment conducted at the catalyst's photothermal reaction temperature (184 °C) without illumination, the CH_4_ yield was only 14% of that achieved under light irradiation. This result clearly indicates that the role of light extends beyond simply heating the catalyst, and actively involves driving the catalytic reaction through photochemical pathways, such as the generation of electron–hole pairs, in synergy with thermal effects. (Figure S13, Supporting Information) Control experiments conducted under visible or infrared‐only illumination (Figure S14, Supporting Information), exhibited significantly lower activities (≈2%) compared to full‐spectrum light. This clearly demonstrates that the photothermal synergy effectively overcomes the thermodynamic barriers and greatly accelerates reaction kinetics.^[^ ^30^ ^]^

Effective separation and transfer of photogenerated charge carriers are crucial for photocatalysis. Time‐resolved photoluminescence (TRPL) decay measurements (Figure 3f) demonstrate that introducing Ni─Ru and Na_inter_ progressively prolongs the charge‐carrier lifetimes. Bare ZrO_2_ shows a fast PL decay with an average lifetime of ≈59.0 ns, characteristic of rapid electron–hole recombination. Deposition of Ni─Ru increases the average lifetime to 80.5 ns, indicating that the bimetallic co‐catalyst facilitates charge separation (electrons can transfer to Ni/Ru sites, reducing recombination). With the 0.2Na─Ni─Ru/ZrO_2_ catalyst, the PL lifetime further extends to 101.7 ns, signifying an even more efficient charge separation in the presence of the Na bridge. Moreover, the TRPL transient exhibits a distinctive inflection at ≈100 ns for the Na‐promoted sample, suggesting the emergence of a new, ultrafast electron transfer pathway. We ascribe this to the Ni^0^─Ni^δ+^─Na_inter_─O─Ru bridge, which provides a conduit for photogenerated electrons to rapidly shuttle between Ni, Ru, and the support. Such a pathway would hasten electron transfer out of the semiconductor into the co‐catalyst and distribute electrons across the multi‐site system, thereby suppressing electron–hole recombination. This efficient charge separation is a key factor enabling more electrons to participate in CO_2_ reduction, underpinning the superior performance of the Na‐bridged catalyst.^[^ ^31^, ^32^ ^]^ Then, we evaluated the CH_4_ production rates at different irradiances and observed a clear non‐linear correlation between catalytic activity and light intensity. This strongly suggests that at higher light intensities, the photothermal effect significantly enhances the direct photochemical reactions.

We also probed CO_2_ and CH_4_ adsorption–desorption behaviors to elucidate how Na_inter_ influences reactant binding and product removal. CO_2_ temperature‐programmed desorption (CO_2_‐TPD, Figure 3g) profiles reveal three types of CO_2_ adsorption on the catalyst surface: a weakly bound physisorbed CO_2_ desorbing ≈93 °C, a moderately strong chemisorbed CO_2_ desorbing at ≈167 °C, and a strongly bound CO_2_ species released between 500–800 °C. Bare ZrO_2_ shows the largest CO_2_ adsorption capacity (especially a huge peak at 167 °C), thanks to abundant basic surface oxygens, but ZrO_2_ alone cannot convert CO_2_ to CH_4_ due to the lack of active hydrogenation sites. Upon adding Ni─Ru, the overall CO_2_ uptake is extensively reduced; in particular, the chemisorbed CO_2_ peak (≈167 °C) diminishes significantly. This suggests that Ni─Ru particles, while providing active hydrogenation sites, covering or altering some of the CO_2_‐binding sites on ZrO_2_, thus limiting CO_2_ adsorption. Interestingly, the 0.2Na─Ni─Ru/ZrO_2_ catalyst recovers some of this lost CO_2_ adsorption ability. The chemisorbed CO_2_ peak is more pronounced with Na, indicating that Na_inter_ introduces new or regenerated CO_2_ binding sites of moderate strength, which are likely important for catalytic turnover. Notably, supplemental experiments indicate that the strongly bound CO_2_ (500–800 °C) is associated mainly with Na_sur_, whereas the moderate chemisorbed CO_2_ is enhanced by Na_inter_ (Figure S15, Supporting Information). In other words, surface Na can bind CO_2_ very strongly, perhaps as unreactive carbonates, but the interfacial Na promotes an optimal chemisorption that is conducive to reaction. Complementary CH_4_‐TPD (Figure 3h) shows that in the high‐temperature range (450–800 °C), 0.2Na─Ni─Ru/ZrO_2_ exhibits the weakest CH_4_ adsorption, in contrast to Ni─Ru/ZrO_2_ which retains more CH_4_. This implies that CH_4_, once formed on 0.2Na─Ni─Ru/ZrO_2_, desorbs more readily from the catalyst surface (lower product binding), which helps prevent product inhibition and ensures active sites free for continuous turnover. Thus, Na_inter_ strikes a beneficial balance: improving CO_2_ adsorption while easing CH_4_ desorption.^[^ ^31^, ^33^, ^34^ ^]^ Finally, kinetic analysis underscores the impact of the Na bridge on reaction energetics. From Arrhenius plots (Figure 3i), the apparent activation energy (*E*_a_) for CO_2_ methanation over Ni─Ru/ZrO_2_ is calculated to be 57.4 kJ mol^−1^. Remarkably, the 0.2Na─Ni─Ru/ZrO_2_ catalyst shows a lower *E*_a_ of 42.9 kJ mol^−1^. This ≈15 kJ mol^−1^ reduction in activation barrier is substantial, explaining the much higher reaction rate. Clearly, the interfacial Na bridge not only improves reactant adsorption and charge separation, but also fundamentally lowers the energy barrier for CH_4_ formation, pointing to a modified reaction pathway or transition state stabilization.

### 2.3 Surface Electronic State Tracking

To unravel the synergy between Ni, Ru, and Na_inter_ during photocatalytic reaction, we carried out quasi in situ XPS under reaction conditions, in situ diffuse reflectance infrared Fourier‐transform spectroscopy (DRIFTS) with time resolution, and density functional theory (DFT) calculations. These studies collectively reveal how the alkali metal bridge modulates the reaction mechanism at the molecular level.

**Figure 4:**
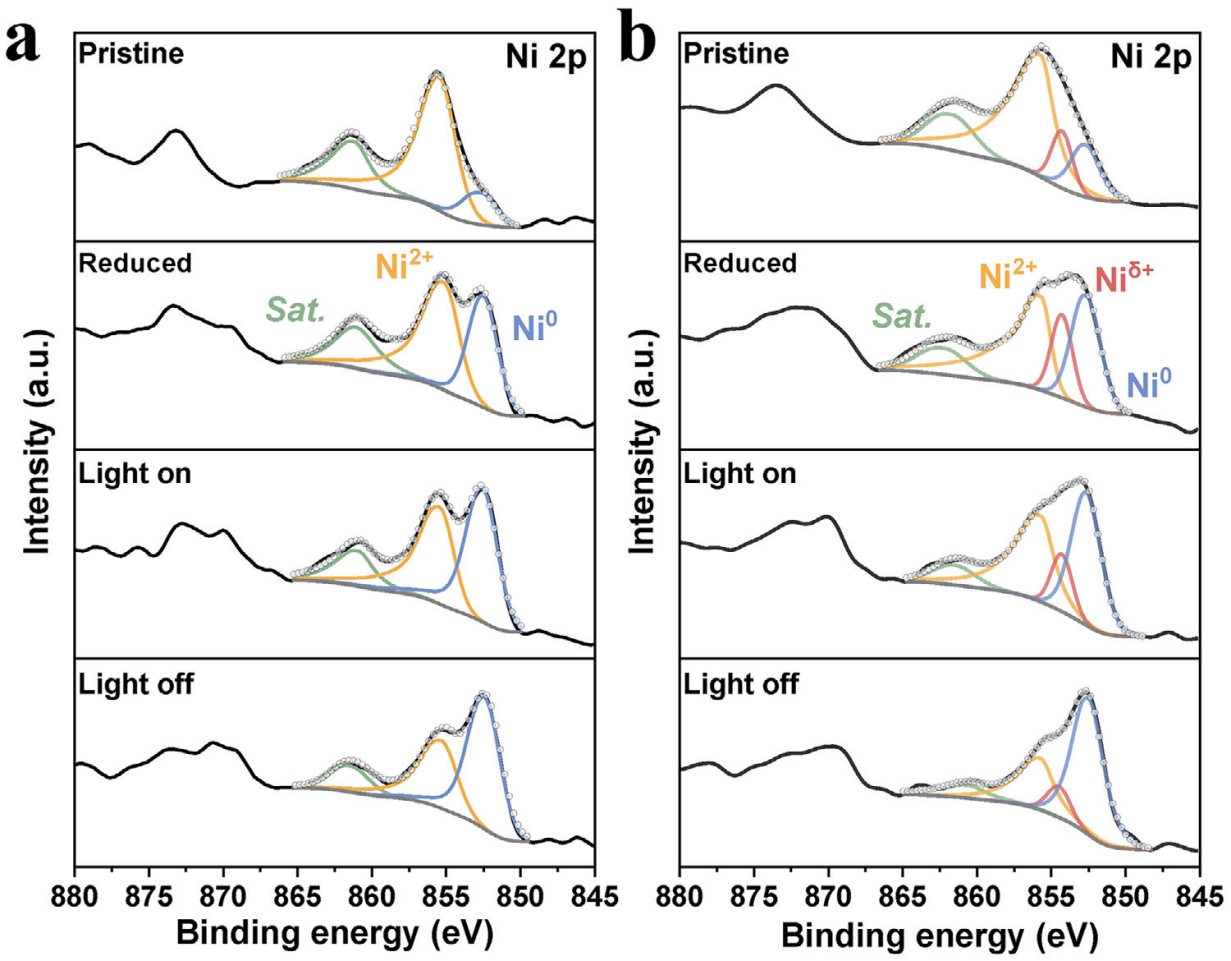
In situ XPS of Ni 2p spectra under reaction‐relevant conditions (initial state, H_2_‐reduced, light on, and light off) for a) Ni‐Ru/ZrO_2_ and b) 0.2Na‐Ni‐Ru/ZrO_2_, highlighting dynamic changes in electronic states during photocatalysis.

First, the Ni 2p spectra of Ni─Ru/ZrO_2_ and 0.2Na─Ni─Ru/ZrO_2_ were measured under different conditions: after H_2_ reduction, during CO_2_ + H_2_ light irradiation, and after photocatalytic reaction (**Figure** **4**a,b). For Ni─Ru/ZrO_2_, H_2_ reduction increases the Ni^0^ signal substantially, as expected, since NiO is partially reduced to metallic Ni (providing active hydrogenation sites).^[^ ^25^ ^]^ During the photocatalytic reaction, the Ni^0^ peak grows further, indicating that Ni remains in a reduced state. This is facilitated by Ru‐driven hydrogen spillover: under reaction conditions, Ru sites dissociate H_2_ and supply atomic H that can migrate to re‐reduce any Ni that begins to oxidize, thus maintaining a high Ni^0^ population.^[^ ^35^, ^36^ ^]^ Simultaneously, the binding energies of Ni 2p and Zr 3d in Ni─Ru/ZrO_2_ shift to higher values during the reaction, whereas the Ru 3d shifts to lower BE (Figure S16; Table S4, Supporting Information). This indicates an accumulation of electron density on Ru (electron‐rich Ru) at the expense of Ni and Zr (which become slightly electron‐deficient) under working conditions. In other words, photogenerated or spillover electrons tend to reside on Ru.^[^ ^37^ ^]^ Such electron redistribution is consistent with Ru being the site for H_2_ activation (an electron‐rich Ru facilitates H─H bond cleavage) and possibly acting as a sink for electrons during CO_2_ activation. The enriched Ru and electron‐deficient Ni/Zr scenario would promote H_2_ dissociation and could polarize CO_2_ adsorbed on Ru, aiding its conversion. The O 1s XPS also shows a small increase in defect oxygen (O_def_) from 52.0% pre‐reaction to 53.2% during reaction, reflecting that the hydrogen‐rich environment (via Ru spillover) slightly reduces the surface, creating more oxygen vacancies in Ni─Ru/ZrO_2_ (Table S5, Supporting Information). In summary, Ni─Ru/ZrO_2_ under reaction conditions benefits from Ru's bifunctional role: providing H spillover to keep Ni active, and hosting electrons that assist in CO_2_ activation, though Ni and Zr do become electron‐poor in the process.

In 0.2Na─Ni─Ru/ZrO_2_, the presence of Na_inter_ leads to distinctly different XPS behavior. Even before reduction, the Ni 2p of 0.2Na─Ni─Ru/ZrO_2_ shows the Ni^δ+^ component, as discussed. After H_2_ reduction, this catalyst has a much higher fraction of Ni^0^ than Ni─Ru/ZrO_2_, confirming that Na_inter_ greatly promotes the reduction of NiO to Ni^0^, consistent with TPR. We also observe that the Ni^δ+^ peak intensity increases after reduction, implying that some Ni atoms at the interface remain in the Ni^δ+^ state even as overall Ni is more reduced. This suggests Na_inter_ accelerates Ru‐mediated hydrogen spillover to Ni and possibly stabilizes a portion of Ni in a δ+ state at the interface. Crucially, during the photocatalytic reaction, the Ni 2p and Zr 3d binding energies in 0.2Na─Ni─Ru/ZrO_2_ remain essentially unchanged compared to the pre‐reaction reduced state (no significant shift in BE). Unlike the Ni─Ru case, we do not see electrons accumulating on Ru at the expense of Ni/Zr; instead, the charge distribution appears more static (Figure S17; Table S6, Supporting Information). We attribute this to the fast and continuous electron flow through the Ni^0^─Ni^δ+^─Na_inter_─RuO_2_ bridge: any electrons introduced (via light excitation or H_2_) are swiftly shuttled between Ni, Na, Ru, and the support, rather than building up on one site (consistent with TRPL). This rapid delocalization of charge likely prevents large chemical shifts in XPS during reaction. In line with this, the Ni^δ+^ species is partially consumed under reaction (its intensity drops) while Ni^0^ grows further, indicating that Ni^δ+^ at the interface gains electrons (reducing to Ni^0^) as the reaction proceeds. This could occur as Ni^δ+^ participates in electron transfer and catalysis, being continuously re‐supplied by Ru's hydrogen spillover.^[^ ^12^ ^]^ Considering the active sites in this study are predominantly metallic surfaces, we propose that the superior H_2_ dissociation capability and H_2_ spillover effect of Ni─Ru are critical factors. The multi‐electron migration facilitated by the Ni─Na_inter_─O─Ru bridge structure may predominantly account for the catalyst's high activity. As shown in Figure 3b,c, the relatively lower activity observed in the initial hour of the reaction likely arises from electron accumulation at the start of the reaction. Once sufficient photogenerated electrons are produced and migrate to active metal surface sites, the electrons and protons together fulfill the reaction's requirement for the eight‐electron/ proton balance, thus significantly enhancing the CO_2_ methanation reaction.

The O 1s spectra for 0.2Na─Ni─Ru reveal a significant jump in O_def_ fraction upon H_2_ reduction (from 52.5% to 61.8%, far higher than the change in Ni─Ru), affirming that Na_inter_ strongly promotes the formation of surface oxygen vacancies and ─OH groups under reducing conditions, consistent with ex situ XPS/EP). However, during CO_2_ hydrogenation, the O_def_ percentage falls from 61.8% to 56.5% (Table S7, Supporting Information). This is a notable contrast to Ni─Ru, where O_def_ rose slightly during reaction. The decrease in O_def_ suggests that many of the oxygen vacancies or defect sites created by Na_inter_ are being consumed or filled during the rapid CO_2_ methanation on 0.2Na─Ni─Ru/ZrO_2_. It is likely that these defects actively participate in CO_2_ activation (e.g., binding oxygen from CO_2_ to form intermediates), and the high reaction rate means they turn over quickly, resulting in a net decrease in detectable O_def_ under steady‐state conditions. Above results shows the raw versus fitted XPS spectra for Ni 2p and O 1s, demonstrating excellent agreement between data and fits, which attests to the reliability of the deconvoluted Ni^0^/Ni^δ+^/Ni^2⁺^ and O_lat_/O_def_ peaks discussed. Taken together, the in situ XPS results depict a scenario where the Na‐bridged catalyst maintains a highly reduced Ni state and a dynamically balanced electron distribution during reaction, thanks to the “electron bridge” facilitating fast electron and hydrogen transfer. This is in stark contrast to the unpromoted catalyst where electrons pile up on Ru. The implication is that Na_inter_ enables Ni and Ru to work in concert without one being electron‐starved – a crucial aspect of enhanced synergy.

### 2.4 Proposed Reaction Pathways

**Figure 5:**
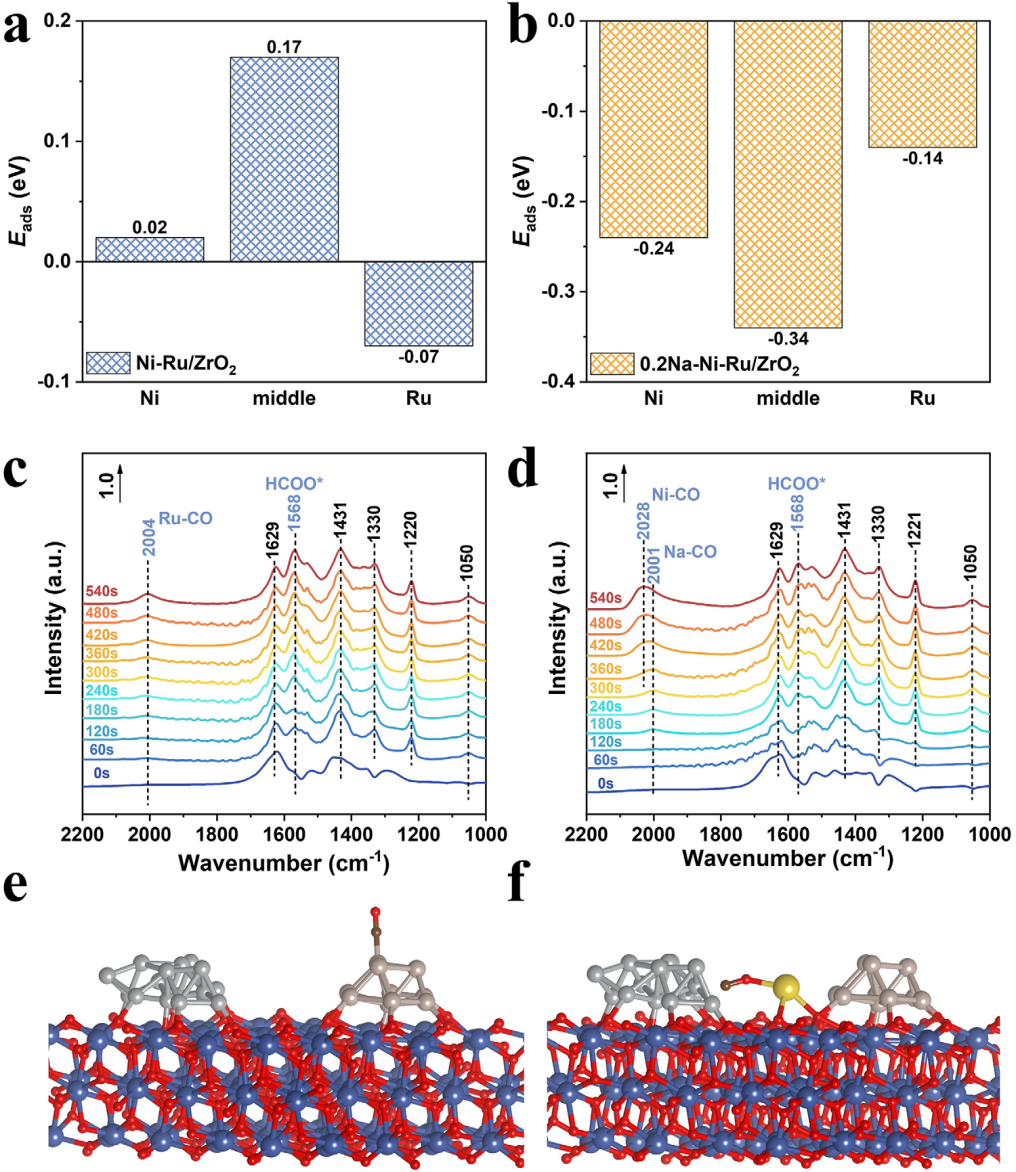
Mechanistic insights from theoretical calculations and in situ DRIFTS spectroscopy. a,b) DFT‐calculated CO_2_ adsorption energies on Ni, Ru in Ni‐Ru/ZrO_2_ and interfacial ZrO_2_/Na sites in 0.2Na‐Ni‐Ru/ZrO_2_ catalysts; c,d) In situ DRIFTS spectra captured during photocatalytic CO_2_ methanation reaction; e,f) Optimized adsorption configurations of CO intermediates on Ni‐Ru/ZrO_2_ and 0.2Na‐Ni‐Ru/ZrO_2_ surfaces, color legend: gray (Ni), brown (Ru), dark blue (Zr), yellow (Na), red (O), black (C).

To further decipher the impact of the alkali metal bridge on the reaction pathway, we combined DFT simulations of key reaction steps with in situ DRIFTS experiments to track surface intermediates. DFT models for Ni─Ru/ZrO_2_ and 0.2Na─Ni─Ru/ZrO_2_ were constructed, with Ni and Ru clusters on ZrO_2_ with and without an interfacial Na at the Ni─Ru junction.^[^ ^13^, ^38^, ^39^, ^40^ ^]^ First, we calculated CO_2_ adsorption energies (**Figure** **5**a,b) on three potential sites: the Ni nanoparticle, the Ru nanoparticle, and the interface between them (the Ni─Ru gap, which for 0.2Na catalyst corresponds to a Na site^[^ ^41^ ^]^). On Ni─Ru/ZrO_2_, CO_2_ adsorption is weakest on Ni, and only mildly exergonic on Ru (–0.07 eV), indicating CO_2_ has a preference to bind (via an O atom) to the Ru surface, albeit very weakly. Adsorption at the Ni─Ru interface (on ZrO_2_ surface between them) is not particularly favorable either in the absence of Na. This aligns with CO_2_‐TPD showing diminished CO_2_ uptake for Ni─Ru. Strikingly, with the Na bridge present, CO_2_ binding strengthens drastically on all sites. Especially, the Na bridging site between Ni and Ru binds CO_2_ with a much more negative free energy (–0.34 eV). This ≈fivefold enhancement in adsorption strength makes the bridged Na site the most favorable anchoring point for CO_2_ in the 0.2Na catalyst. DFT thus supports the experimental observation that Na_inter_ introduces new CO_2_ binding capability (moderate chemisorption) that Ni─Ru lacked. Indeed, the interfacial Na appears to act like a “sodium ion bridge” that polarizes and captures CO_2_, presumably by forming a Na─O(CO_2_) bond.

We then carried out time‐resolved DRIFTS to observe how CO_2_ is activated and converted on the catalyst surface, comparing Ni─Ru/ZrO_2_ and 0.2Na─Ni─Ru/ZrO_2_. For each catalyst, we allowed CO_2_ and H_2_ to adsorb in the dark and then turned on the light to initiate the reaction, collecting IR spectra at set time intervals. During the dark adsorption phase on Ni─Ru/ZrO_2_, we observe bands in the 1000–1650 cm^−1^ region indicative of carbonate and bicarbonate species: peaks at 1297, 1522, and 1629 cm^−1^ can be assigned to various surface carbonates, while features at 1220 and 1297 cm^−1^ correspond to monodentate bicarbonate (HCO_3_^−^) species (Figure S18a, Supporting Information).^[^ ^20^, ^42^ ^]^ In the O─H stretching region, a broad band below 3500 cm^−1^ is seen (O─H of bicarbonate), and sharp peaks at 3590–3724 cm^−1^ appear, which are attributed to surface ─OH groups on ZrO_2_.^[^ ^43^, ^44^ ^]^ The intensity of these ─OH signals correlates with CO_2_ adsorption capacity (more CO_2_ adsorption leads to more surface bicarbonate and associated ─OH). When illumination begins, significant changes occur in the Ni─Ru/ZrO_2_ DRIFTS spectrum. The bicarbonate O─H band (<3500 cm^−1^) diminishes rapidly, indicating that these HCO_3_^−^ species are being consumed or converted under reaction conditions. Concurrently, new peaks emerge that signify key reaction intermediates: a peak at 1568 cm^−1^ grows, assignable to formate species (HCOO*) bound to the surface, and a peak at 2004 cm^−1^ develops, which corresponds to linearly adsorbed CO (CO* atop a metal site). Given our DFT models, we associate the 2004 cm^−1^ band with CO adsorbed on a Ru site (bridging or atop Ru) since DFT‐calculated spectra show a Ru─CO stretch consistent with this frequency (Figure 5c). Later in the reaction (after continued illumination), bands at 2967 and 2886 cm^−1^ appear, which are attributed to asymmetric and symmetric C─H stretch vibrations of surface methane‐like species (–CH_3_ or –CH_2_–, denoted as CH_x_*).^[^ ^20^, ^25^, ^45^ ^]^ The presence of these bands confirms that CO_2_ is indeed being converted all the way to methane on Ni─Ru/ZrO_2_, passing through formate and CO intermediates. Importantly, in the Ni─Ru case we did not detect a distinct IR band for CO adsorbed on Ni (which would typically appear in the 2020–2050 cm^−1^ region if Ni─CO were present). This absence suggests that once CO is formed (on Ru), it likely migrates to Ni and is rapidly hydrogenated to CH_4_ without accumulating as a detectable Ni─CO species. This implies that the hydrogenation of CO to methane on Ni is relatively fast, whereas the prior step (formate to CO on Ru) might be the slower, rate‐limiting step. Indeed, Ni was calculated to bind CO more strongly than Ru (DFT: CO binding energy –2.84 eV on Ni vs –2.64 eV on Ru), making thermodynamically favorable for CO to move from Ru to Ni (Figure S19a, Supporting Information). The kinetic bottleneck in Ni─Ru/ZrO_2_ appears to be forming that CO in the first place (i.e., overcoming the formate intermediate on Ru). Another observation is that Ru in Ni─Ru/ZrO_2_ is serving a dual function: activating CO_2_ and dissociating H_2_. The necessity for Ru to handle both tasks could crowd the Ru surface or divert some Ru sites to hold H atoms, potentially slowing the formate‐to‐CO conversion (since it requires adjacent CO_2_ and H on Ru). This mechanistic picture from DRIFTS aligns with our kinetic data that Ni─Ru without Na has a higher activation energy, hinting at a less efficient pathway.

With the Na‐bridged 0.2Na─Ni─Ru/ZrO_2_, the DRIFTS signatures of intermediates change in a way that corroborates the altered pathway predicted by DFT. During the CO_2_ adsorption dark phase on 0.2Na─Ni─Ru/ZrO_2_, we see similar carbonate and bicarbonate features as Ni─Ru, but notably the intensity of the carbonate band (e.g., at 1459 cm^−1^) is slightly reduced. This suggests that the presence of Na_inter_ partially suppresses the formation of strongly bound carbonates on ZrO_2_, likely because some CO_2_ is instead captured at the Na sites (as indicated by DFT) rather than all converting to carbonate species on the support. Once the reaction is initiated by light, the same key intermediates appear – formate and CO─ but their behavior is distinctly different. A CO band arises at ≈2001 cm^−1^ early in the reaction, which we assign to CO adsorbed on the Na species site (Na─CO). This assignment is supported by DFT, which shows that CO binding on the Na bridge yields a characteristic frequency ≈2001 cm^−1^ (Figure 5d). Remarkably, as the reaction proceeds (within the first 300–400 s of illumination), this CO band gradually shifts and broadens, moving to ≈2028 cm^−1^. By the 360 s mark, the dominant CO band is centered at ≈2028 cm^−1^, which corresponds to CO adsorbed on Ni (Ni─CO) sites. This time‐resolved shift provides direct evidence that CO produced at the Na interface site is transferring to the Ni surface. In tandem with this, the formate peak at 1568 cm^−1^ is significantly less intense for 0.2Na─Ni─Ru/ZrO_2_ than for Ni─Ru, indicating that formate is consumed much faster when Na_inter_ is present. These observations fit a mechanism where CO_2_ is initially activated at the Na bridge forming a Na─OCO or Na─HCOO* intermediate, which then quickly receives H to form CO that migrates to Ni. The rapid loss of formate signal implies that the formate‐to‐CO conversion, previously slow on Ru, is now greatly accelerated at the Na site. This is likely due to the “electron bridge” effect: the Ni^0^─Ni^δ+^─Na─RuO_2_ ensemble can swiftly shuttle electrons to facilitate the cleavage of the C─O bond in formate, expelling an ─OH (which could combine with H to form H_2_O) and leaving CO bound to Na. DFT calculations confirm that the Na‐mediated formate conversion requires a much smaller energy input (1.76 eV vs 2.86 eV on Ru, discussed below). Once CO is formed and migrates to Ni (as evidenced by the Ni─CO band at 2028 cm^−1^), it undergoes hydrogenation on Ni to yield methane, similar to the Ni─Ru case for the final step. However, thanks to the faster CO formation step, CO accumulates slightly on Ni in the Na‐promoted catalyst (hence we actually detect Ni─CO transiently, whereas we did not in Ni─Ru). Importantly, in this Na‐bridged mechanism Ru's role is simplified: Ru now mainly acts as a hydrogen supplier. With CO_2_ activation happening at Na and subsequent steps on Ni, Ru is free to dissociate H_2_ continuously, providing a steady flux of spillover H (H*) to the nearby Na and Ni sites. This division of labor means Ru no longer needs to hold formate or CO_2_ – alleviating the site competition that likelyx hindered the Ni─Ru catalyst. Additionally, the presence of abundant spillover H maintains a highly reduced local environment, as observed in XPS with Ni staying reduced and many oxygen vacancies created and then utilized. In summary, the DRIFTS and DFT evidence together illustrate a new reaction pathway in the Na‐bridged catalyst: CO_2_ is predominantly activated at the Na bridge as formate, quickly converted to CO with the help of rapid electron transfer, and the CO intermediate shuttles to Ni for final hydrogenation to CH_4_. Ru provides hydrogen but is not overloaded with dual functionalities. This cooperative mechanism drastically lowers the kinetic barriers of the slow steps and enhances overall efficiency.

**Figure 6:**
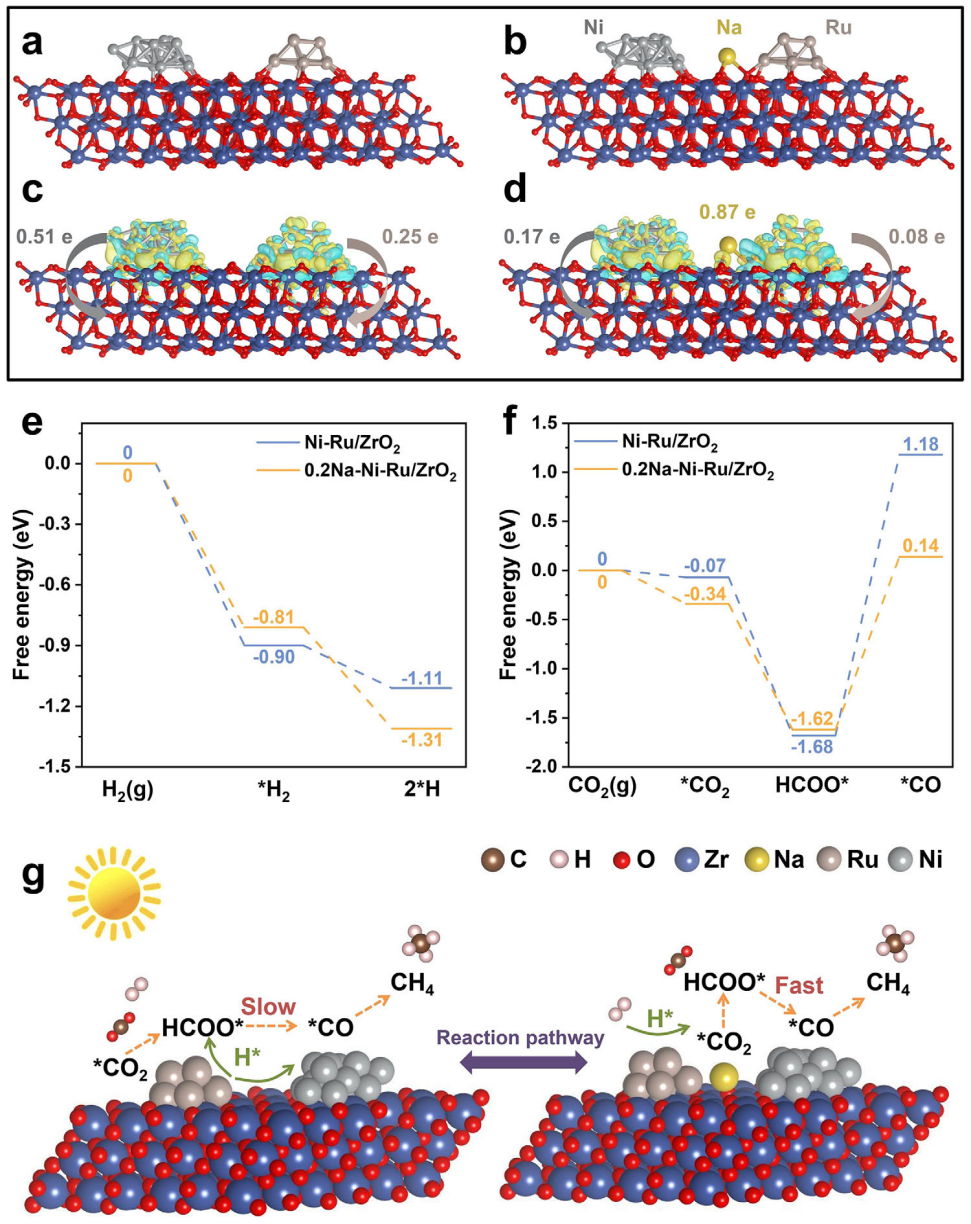
DFT‐derived electronic structure and reaction energetics. a,b) Optimized structural models (front view) of Ni‐Ru/ZrO_2_ and 0.2Na‐Ni‐Ru/ZrO_2_; c,d) Charge density difference plots indicating electron density accumulation (yellow) and depletion (cyan); e,f) Gibbs free energy diagrams for key catalytic steps (H_2_ dissociation and CO_2_ activation); g) Reaction pathway comparison illustrating lowered activation barriers and improved kinetics via interfacial Na bridging in the 0.2Na‐Ni‐Ru/ZrO_2_.

The proposed mechanisms are further supported by calculated Gibbs free energy profiles for the crucial steps of H_2_ dissociation and CO_2_ reduction on both catalysts (**Figure** **6**e,f). On Ni─Ru/ZrO_2_, Ru is the preferred site for H_2_ splitting (as expected, since Ni alone is less effective at dissociating H_2_). This step is exergonic by ≈1.11 eV, i.e., Ru─H formation is thermodynamically favorable. In 0.2Na─Ni─Ru/ZrO_2_, Ru still serves as the H_2_ dissociation site, but interestingly the process releases ≈1.31 eV, indicating an even stronger driving force (likely because the electron‐rich environment from Na makes H─H cleavage and adsorption more facile). Thus, the Na bridge enhances the creation of active H species on Ru. For CO_2_ activation, the pathway on Ni─Ru/ZrO_2_ (Figure 6f) begins with CO_2_ adsorption on Ru (via an O atom, forming a bent CO_2_^δ−^ species on Ru). This adsorption is only slightly favorable (Δ*G* = –0.07 eV) as noted. When a surface H (from dissociated H_2_ on Ru) adds to this CO_2_, a formate (HCOO*) species is formed on Ru. This step is exergonic by 1.55 eV, reflecting a significant stabilization of formate on Ru (in line with the strong formate DRIFTS band we observed). The sizable exotherm also suggests that formate, once formed, is quite stable on Ru. However, the next step – conversion of formate (HCOO*) to a CO intermediate (likely via HCOO* + H* → CO* + H_2_O on Ru) – is highly endergonic, requiring +2.86 eV. This large uphill energy corresponds to breaking the C─O bond of formate and desorbing an ─OH (which joins another H to form H_2_O). The 2.86 eV energy penalty explains why formate tended to accumulate on Ni─Ru and why CO formation was the bottleneck. Such a high barrier severely limits the overall reaction rate at this step, consistent with high measured *E*_a_.

In contrast, on 0.2Na─Ni─Ru/ZrO_2_, CO_2_ adsorption at the Na site is significantly more favorable (ΔG = –0.34 eV), meaning CO_2_ is readily captured. The formation of a formate species bound to Na (Na─OCOH, with H likely coming from a Ru─H) is exergonic by 1.28 eV. This is slightly less exothermic than on Ru (1.28 vs 1.55 eV), possibly because the formate is not as strongly bound to Na as it was to Ru, which actually is beneficial – it implies the formate is more reactive. Indeed, the subsequent step to produce CO (Na─CO + OH) requires only +1.76 eV, which is a drastically lower energy hurdle than 2.86 eV. Thus, the highest barrier in the Na‐bridged pathway is much reduced. Thermodynamically, after this step the Na─CO intermediate is formed (and an ─OH likely on a nearby site or recombined into water). As the final steps, CO will relocate to Ni and hydrogenate to CH_4_. Our DFT did not explicitly calculate the CO migration and hydrogenation on Ni, but given that Ni binds CO strongly and can hydrogenate CO (steps that are known to be facile on Ni under these conditions), we expect those steps to be downhill or have manageable barriers. The critical point is that by rerouting CO_2_ through the Na site, the formate‐to‐CO conversion is no longer a show‐stopping endergonic step; it becomes surmountable under reaction conditions, especially considering photo‐excitation can supply additional energy. This thermodynamic analysis perfectly complements the DRIFTS findings: the Na‐mediated route is energetically more favorable, leading to faster formate turnover and CO production, which in turn accelerates CH_4_ formation – consistent with the lower apparent E_a_ measured for 0.2Na─Ni─Ru. In essence, Na_inter_ opens a new low‐energy pathway for the reaction that bypasses the kinetic trap on Ru.

The role of the Na bridge in tuning electronic structure was also visualized via Bader charge and charge density difference analyses (Figure 6a–d). In Ni─Ru/ZrO_2_, we find that the Ni─Ru bimetallic particles donate a total of ≈0.76 *e^−^* to the ZrO_2_ support (electrons flow into the support, possibly into surface O or defect states). With the interfacial Na present, the total charge transferred to the support increases to ≈1.12 *e^−^*. Remarkably, of this, Na itself carries ≈ 0.87 *e^−^* (meaning Na becomes negatively polarized, as Na^δ−^). This indicates that Na is the primary conduit for electron transfer: it draws electron density from the Ni─Ru and channels it toward the support (and also holds some electron density localized). In doing so, Na effectively connects the metal particles and support in an electronic network. The charge density difference plots (Figure 6c,d) illustrate that in 0.2Na─Ni─Ru/ZrO_2_, there is a pronounced build‐up of electron density around the Na at the interface (yellow regions denote electron accumulation). This enriched electron cloud around Na is likely what enhances CO_2_ adsorption and activation, as anionic or electron‐rich sites can better stabilize the electron‐poor CO_2_ molecule (polarizing it into CO_2_^δ−^ for easier reduction).^[^ ^46^ ^]^

Additionally, we see that the Ni surface in Ni─Ru/ZrO_2_ suffers electron depletion (cyan regions on Ni, indicating Ni lost electron density to Ru and support). In the Na‐bridged system, however, the Ni nanoparticle retains a more balanced electron distribution (far less cyan on Ni, and some yellow indicating slight electron gain) (Figure S20, Supporting Information). This suggests that Na prevents Ni from becoming overly electron‐deficient by rerouting some electron density. A better electron density on Ni is beneficial for the CO hydrogenation steps, since Ni can donate electrons to adsorbed CO and H easier. Meanwhile, Ru in 0.2Na─Ni─Ru is bonded to O (as RuO_2_‐like), which withdraws some electron density from Ru. But because Na is supplying electrons to that region, RuO_2_ is electronically connected to Ni via Na, forming the Ni─Na─RuO_2_ bridge where electrons can flow dynamically. These electronic structure changes underscore that the alkali metal bridge is not a passive spectator ion; it actively reshapes the electronic landscape of the catalyst. By accumulating electrons and mediating their distribution, Na enables Ni and Ru to synergistically share the tasks of activation and hydrogenation without one site monopolizing electrons from the other.^[^ ^10^ ^]^

Based on all the above findings, we can now articulate the cooperative mechanism in the Na‐bridged Ni─Ru catalyst. The addition of a small amount of Na at the Ni─Ru interface creates an electron‐rich alkali bridge (Ni^0^─Ni^δ+^─Na^δ−^─RuO_2_) that connects the metal nanoparticles and the support. Under reaction conditions, Ru (in proximity to Na) efficiently splits H_2_ into H atoms, which migrate to Ni and the Na interface. CO_2_ is predominantly adsorbed and activated at the Na^δ−^ site, forming a reactive formate intermediate that is rapidly converted to CO, thanks to the facile electron transfer through the bridge. The CO intermediate, in turn, migrates to the Ni surface, where it is promptly hydrogenated to CH_4_. Ru's role is largely to continuously provide hydrogen and maintain a reducing environment, and any electron exchange needed for these steps is shuttled via the Na bridge – preventing charge build‐up or depletion at individual sites. In this way, Ni, Ru, and Na work in concert: Ni and Ru alone had difficulty synergizing fully, but Ni, Na, and Ru together form a three‐component team that overcomes the limitations of the binary system. This strategy leads to a remarkable enhancement in catalytic performance (an order‐of‐magnitude rate increase and high stability), and a fundamentally improved reaction pathway with lower energy barriers. The alkali metal bridge not only boosts activity but also alters the mechanism to achieve deep synergy between Ni and Ru that was previously elusive.

## 3 Conclusion

In summary, we have successfully demonstrated that constructing interfacial alkali‐metal bridges significantly enhances the photocatalytic performance of Ni─Ru/ZrO_2_ catalysts for CO_2_ methanation. By deliberately anchoring Na species at the Ni─Ru and ZrO_2_ interface and subsequently removing surface Na, we established a robust Ni^0^─Ni^δ+^─Na_inter_─O─Ru electronic bridge, greatly improving electron transfer, intermediate stabilization, and charge carrier lifetime. Structural and spectroscopic analyses clarified the critical role of interfacial Na, revealing that this strategy not only promotes effective charge delocalization across metal nanoparticles and the support but also facilitates optimal adsorption–desorption dynamics for the key intermediates. Mechanistic studies combining in situ DRIFTS and DFT calculations elucidated that the interfacial Na drastically reduces the activation energy barrier for the rate‐determining formate‐to‐CO step, rerouting the reaction pathway and significantly boosting CH_4_ formation kinetics. The resulting catalyst exhibits outstanding photocatalytic activity (1882.7 µmol·g^−1^·h^−1^) and remarkable stability, offering compelling proof of concept for alkali‐metal bridging as a powerful tool for designing high‐performance photocatalysts. This interfacial engineering approach holds promise for broad applications in catalytic systems requiring precise multi‐site synergy.

## Conflict of Interest

The authors declare no conflict of interest.

## Supporting information



Supporting Information

## Data Availability

The data that support the findings of this study are available in the supplementary material of this article.;
