# Supplementary material for: Strategically Constructing Alkali‐Metal Interfacial Bridges to Boost Photocatalytic CO2 Methanation on Supported Ni─Ru Bimetallic Catalysts
Source: Adv Sci (Weinh). 2025 Aug 4;12(40):e09454. doi: 10.1002/advs.202509454 (PMC12561269; doi:10.1002/advs.202509454)
Supplement: Supplementary file 1 — Supporting Information [file ADVS-12-e09454-s001.docx]

Supporting Information

**Strategically Constructing Alkali-Metal Interfacial Bridges to Boost Photocatalytic CO_2_ Methanation on Supported Ni–Ru Bimetallic Catalysts**

Xiaolei Guo,^[a,b, †]^ Yuqi Wu,^[a^^,†]^ Shengrong Zhou,^[b]^ Yuhang Shao,^[b]^ Yasuo Izumi,*^,[c]^ Jinlu He*^,[a]^ and Hongwei Zhang*^,[b]^

^a^ College of Chemistry and Chemical Engineering, Inner Mongolia University, Hohhot 010021, PR China

^b^ Key Laboratory of Development and Application of Rural Renewable Energy, Biogas Institute of Ministry of Agriculture and Rural Affairs, Chengdu 610041, PR China

^c^ Department of Chemistry, Graduate School of Science, Chiba University, Yayoi 1-33, Inage-ku, Chiba 263-8522, Japan

^†^ X. Guo and Y. Wu contributed equally to this work.

**1. Experiment:**

**1.1. Material：**

Nickel chloride hexahydrate (NiCl_2·_6H_2_O, 99%) and anhydrous ruthenium trichloride (RuCl_3_, Ru content 45-55%) were purchased from Shanghai Maclin Biochemical Technology Co. Sodium nitrate (NaNO_3_, AR) was purchased from Chengdu Cologne Chemical Co. ZrO_2_ (JRC-ZRO-3; predominantly monoclinic, followed by tetragonal, specific surface area 94.4 m^2^/g) was obtained from the Japan Society of Catalysis. All reagents were not purified before use.

**1.2. xNa-Ni-Ru/ZrO_2_ synthesis:**

The xNa-Ni-Ru/ZrO_2_ catalyst was prepared using an impregnation method. First, a measured amount of ZrO_2_ was dispersed in 20 mL of deionized water, ultrasonicated for 5 min, and stirred at 300 rpm for 30 min to achieve uniform dispersion. Subsequently, a specific volume of NaNO_3_ solution (0.1 M) was added, followed by continued stirring for 30 min. Appropriate amounts of NiCl_2_ (1 M) and RuCl_3_ (0.1 M) solutions were then introduced to achieve 10 wt% and 1.5 wt% loadings, respectively. The mixture was stirred for 1 h before being heated to 80 °C to evaporate the solvent completely. The resulting sample was thoroughly ground and reduced under a 10% H_2_/Ar atmosphere at 450 °C. 0.2Na-Ni-Ru/ZrO_2_ as example, to obtain a Na : Ni molar ratio of 0.2, we added 1.022 mL of 0.1 M NaNO_3_ solution (equivalent to 0.1022 mmol Na⁺). For catalysts with other Na loadings (0.05–1.0 wt%), the amount of NaNO_3_ solution was adjusted accordingly to maintain the desired ratios. To remove excess surface Na, the reduced sample was filtered and washed with 1 L of deionized water. The obtained powder was dried overnight at 80 °C and re-reduced under the same 10% H_2_/Ar atmosphere at 450 °C. The final product was denoted as xNa-Ni-Ru/ZrO_2_ (x = 0.05, 0.1, 0.2, 0.4, 0.6, 0.8, 1.0, where x represents the molar ratio of Na to Ni).

**1.3. Synthesis of xNa-Ni-Ru/ZrO_2_-NW:**

The xNa-Ni-Ru/ZrO_2_-NW catalyst was prepared following a procedure analogous to Section 1.2, with the exception that the final catalyst was obtained directly after the first reduction step, omitting the extensive deionized water washing process.

**1.4. Synthesis of Ni-Ru/ZrO_2_:**

The Ni-Ru/ZrO_2_ catalyst was prepared via an impregnation method. A measured amount of ZrO_2_ was dispersed in 20 mL of deionized water, ultrasonicated for 5 min, and stirred at 300 rpm for 30 min to ensure uniform dispersion. Appropriate volumes of NiCl_2_ (1 M) and RuCl_3_ (0.1 M) solutions were then added to achieve 10 wt% and 1.5 wt% loadings, respectively. The mixture was stirred for 1 h and subsequently heated to 80 °C until complete solvent evaporation. The final photocatalyst was obtained by grinding the dried sample and reducing it under a 10% H_2_/Ar atmosphere at 450 °C.

**1.5. Synthesis of Ni/ZrO_2_:**

The Ni/ZrO_2_ catalyst was prepared following a procedure analogous to Section 1.4, with the omission of the RuCl_3_ solution addition.

**1.6. Synthesis of Ru/ZrO_2_:**

The Ru/ZrO_2_ catalyst was prepared following a procedure analogous to Section 1.4, with the omission of the NiCl_2_ solution addition.

**2. Characterization:**

The prepared powders were analyzed by X-ray photoelectron spectroscopy (XPS) using a Thermo Scientific K-Alpha system to determine surface binding energies. All peaks were energy-calibrated relative to the C 1s peak of amorphous carbon at 284.8 eV. In-situ XPS measurements for Ni-Ru/ZrO_2_ and 0.2Na-Ni-Ru/ZrO_2_ were conducted on a Thermo Escalab 250Xi to analyze surface binding energy changes before/after reduction and reaction. Morphological details were observed using a FEI Talos F200x transmission electron microscope (TEM), with HAADF analysis performed using a Super-X EDX detector. Aberration-corrected scanning transmission electron microscopy (STEM) was carried out on a JEM-ARM200F at 200 kV. X-ray diffraction (XRD) analysis was performed on a Rigaku Ultima IV diffractometer with Cu Kα radiation. The Ni, Ru, and Na contents in the reduced catalysts were quantified by inductively coupled plasma optical emission spectroscopy (ICP-OES) on a PE Avio 200. Optical properties were measured using a UV-3600I Plus ultraviolet-visible-near-infrared spectrophotometer. Electron paramagnetic resonance (EPR) tests for oxygen vacancy analysis were conducted on a BRUKER EMXPLUS. H_2_-TPR, CO_2_-TPD, and CH_4_-TPD analyses were performed on a BelCata II，and prior to TPR and TPD, all samples were subjected to the same reduction protocol used in catalyst preparation (450 °C, 10 % H_2_/Ar) to ensure a uniform starting state that mirrors the catalyst’s activated form. The reduced powders were then stored in air before each characterisation experiment. *In-situ* DRIFTS during adsorption and reaction stages was monitored using a Bruker Tensor II. Surface temperature under light irradiation was detected with a FLIR infrared thermal imaging camera. Time-resolved photoluminescence (TRPL) measurements were conducted on an FLS-1000 transient fluorescence spectrometer with a 350 nm excitation wavelength.

y=A+B_1_exp(−*t*/τ_1_)+B_2_exp(−*t*/τ_2_) （1）

**3.** **Photocatalytic performance evaluation:**

The reaction was conducted in a Perfect 6A glass circulation system (100 mL) and a U-shaped quartz reactor (45 mL). A 300 W Xenon lamp (CEL-HXUV300-T3, Beijing China Education AuLight Technology Co., Ltd.) served as the light source, maintaining a constant light intensity of 1.5 W cm^-2^. For each test, 20 mg of photocatalyst was placed in the U-shaped quartz reactor and evacuated to 10^-2^ Pa using a rotary pump for 1 hour. Subsequently, the catalyst was *in-situ* reduced under a 10% H_2_/Ar atmosphere at 450 °C for 30 minutes, followed by immediate evacuation of the reactor for 5 minutes while hot to prevent gas adsorption. The reactor was then filled with a mixed reaction gas at 64 kPa (5% CO_2_, 45% H_2_ and 50% Ar by volume), and the reaction was initiated under light irradiation. The selected CO_2_:H_2_ ratio was intended to ensure complete CO_2_ conversion within the sealed reaction system and to continuously maintain the active state of the metallic catalytic surface sites, consistent with our previously reported experimental conditions. Additionally, Ar was introduced as a balance gas primarily for safety considerations. Reaction products were analyzed by an online gas chromatograph (GC9720II, Fuli Instruments, China) using Ar (0.40 MPa, purity >99.999%) as the carrier gas.  Quantitative analysis was performed with a flame ionization detector (FID) and a thermal conductivity detector (TCD). We also performed thermal catalytic CO_2_ methanation experiments under identical gas composition and catalyst conditions (0.2Na–Ni–Ru/ZrO_2_), maintaining the reaction temperature at approximately 184°C through external heating without illumination. Wavelength-dependent tests achieved by inserting optical cutoff filters, we observed the activities that under visible (400–800 nm) and infrared (>800 nm) light alone.

In this study, TON is defined as the ratio of the moles of CO_2_ converted to CH_4_ to the total moles of active metal sites in the reactor:

$$TON= \frac{n_{CH4}}{n_{\mathrm{metal}}}$$

For simplicity, we considered all Ni, Ru, and Na atoms in the catalyst as potential active sites. Although not all atoms are surface-exposed (thus making this calculation a lower-bound estimate), it provides a straightforward and practical metric.

Taking the optimized 0.2Na–Ni–Ru/ZrO_2_ catalyst as an example, the catalyst loading was 20 mg, comprising approximately 10 wt% Ni (~2 mg Ni, equivalent to about 0.034 mmol Ni), 1.5 wt% Ru (~0.3 mg Ru, equivalent to about 0.003 mmol Ru), and 0.045 wt% Na (~0.009 mg Na, equivalent to about 0.0004 mmol Na). Summing these components, the total active metal sites were approximately 0.0374 mmol (or 3.74×10⁻^5^ mol).

Under typical reaction conditions (as shown in Fig. 3a), CH_4_ production over 2 hours was approximately 1882.7 µmol·g⁻^1^·h⁻^1^ × 0.02 g × 2 h ≈ 75.3 µmol CH_4_, corresponding to 75.3 µmol of CO_2_ converted. To remain consistent with the cycling tests, we measured the cumulative CH_4_ produced after 4 hours, obtaining approximately 158.8 µmol (the actual measured value was approximately 159 µmol). Thus, using this 4-hour data, representing more complete conversion conditions, we calculated the TON as follows:

$$TON= \frac{159 \mu mol}{3186 \mu mol （metal atoms）}=0.05$$

This indicates that about 5% of the active metal atoms participated in the CO_2_ conversion event under these conditions.

**4. DFT Calculation：**

DFT calculations were performed using the projector augmented wave (PAW) method with the Vienna ab initio Simulation Package (VASP) simulation package.^[1]^ The interaction between the ionic cores and the valence electrons are described with the PAW method.^[2]^ The exchange and correlation interactions are described using the Perdew-Burke-Ernzerh of generalized gradient approximation (GGA).^[3]^

The cut-off energy is set to 400 eV for all calculations, and the convergence criterion of energy and force are 10^-4^ eV and 0.001 eV/Å, respectively. Besides, a 1× 1× 1 Gamma-centered k-point mesh is used for optimization of geometric structure and other calculations of the Ni-Ru/ZrO_2_ and Na-Ni-Ru/ZrO_2_. The DFT model employed in this study (Ni_16_Ru_10_ cluster supported on ZrO_2_ (111) with an interfacial Na atom) was specifically designed based on experimental findings. Although the Ni:Ru atomic ratio in the model (approximately 1.6:1) differs from the experimental catalyst (approximately 7:1 with Ni_10_Ru_1.5_), this simplification was necessary due to computational constraints. The chosen model effectively captures the key structural and electronic features observed experimentally—specifically, the interfacial Na bridging structure (Ni^0^–Ni^δ+^–Na_inter_–O–Ru) suggested by our XAFS, HR-TEM and XPS results. Despite the ratio difference, this approach accurately reproduces the experimental trends and the essential physical interactions relevant to catalytic performance, ensuring a valid representation of the catalytic system. To obtain the relaxed ZrO_2_, Ni_16_ and Ru_10_ surface structure from which to build the Ni_16_-Ru_10_-ZrO_2_ interface, the units were cut along the direction of the ZrO_2_ bulk planes (111). We added a 15 Å vacuum layer along the c-axis, which is necessary to consider the influence of the periodic boundary conditions. Meanwhile, the Ru_10_ and Ni_16_ cluster was built and placed on the ZrO_2_ (111) surface. The DFT+U method was employed in the present study, where a Hubbard type correction was applied on the 4d orbitals of Zr and 3d orbitals of Ru and Ni. We set U_eff,Zr_= 4.0 eV for Zr 4d ^[3]^ orbital U_eff,Ru_= 3.0 eV and U_eff,Ni_= 5.5 eV for Ru and Ni 3d orbital.^[5,6]^

Finally, the adsorption energies ($E_{ads}$)were calculated as:

$$E_{ads}=E_{ad/sub}-E_{ad}-E_{sub}$$

where $E_{ad/sub}$is the total energies of the optimized adsorbate/substrate system, $E_{ad}$ is the energies of adsorbate in the gas phase, and $E_{sub}$ is the energies of clean substrate, respectively.

The free energy (ΔG) for elemental reaction step were calculated as:

$$\Delta G=\Delta E + {\Delta E}_{ZPE}-T\Delta S$$

where Δ*E* is the difference between the total energy, ${\Delta E}_{ZPE}$ and Δ*S* are the differences in the zero-point energy and the change of entropy, T is the temperature (T=298.15 K in this work), respectively.

Fig S1. XRD patterns of the series of catalysts xNa-Ni-Ru/ZrO_2_.


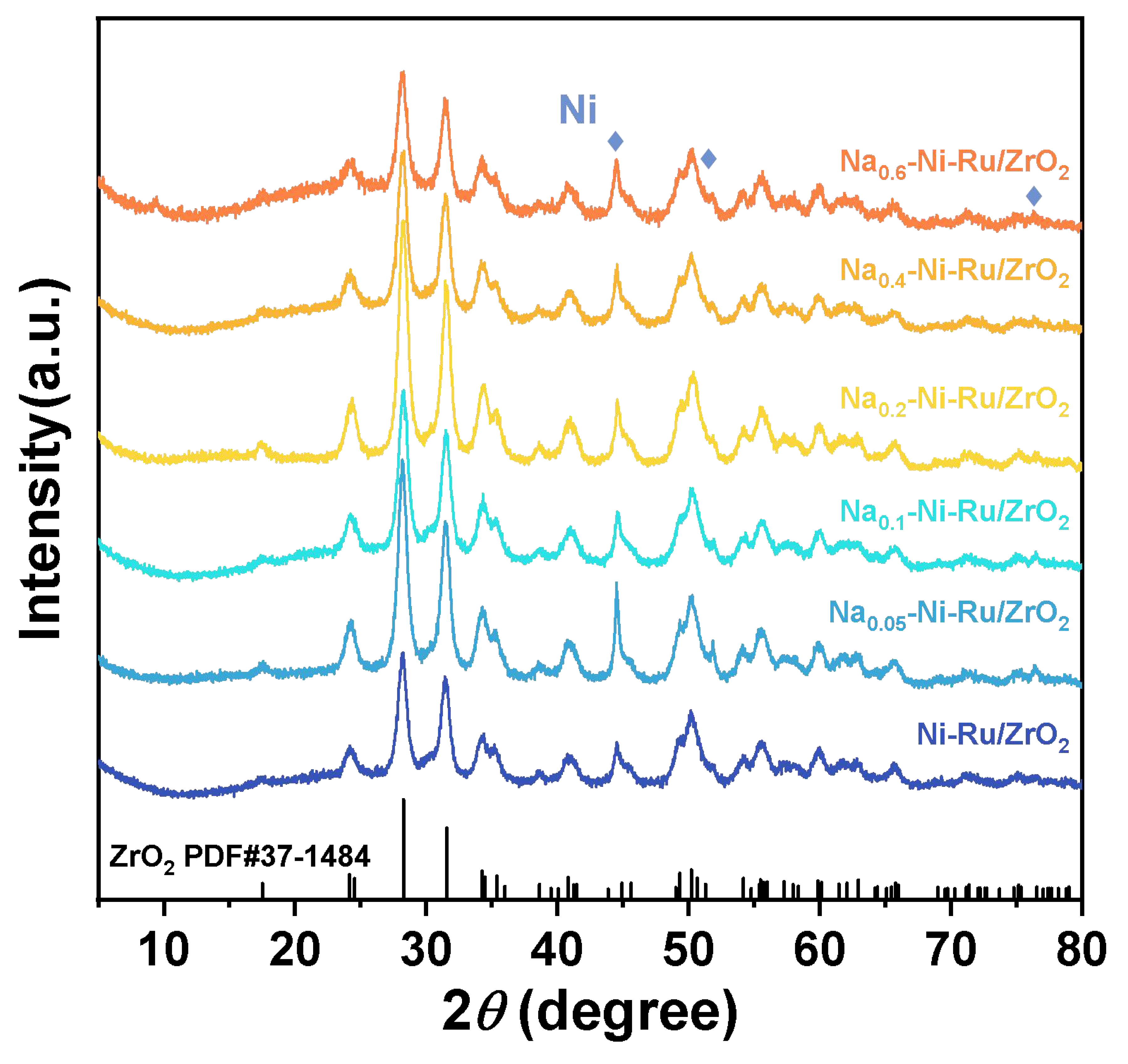


Fig S2. H_2_-TPR of Ni/ZrO_2_, Ru/ZrO_2_ and Ni-Ru/ZrO_2_ photocatalysts.


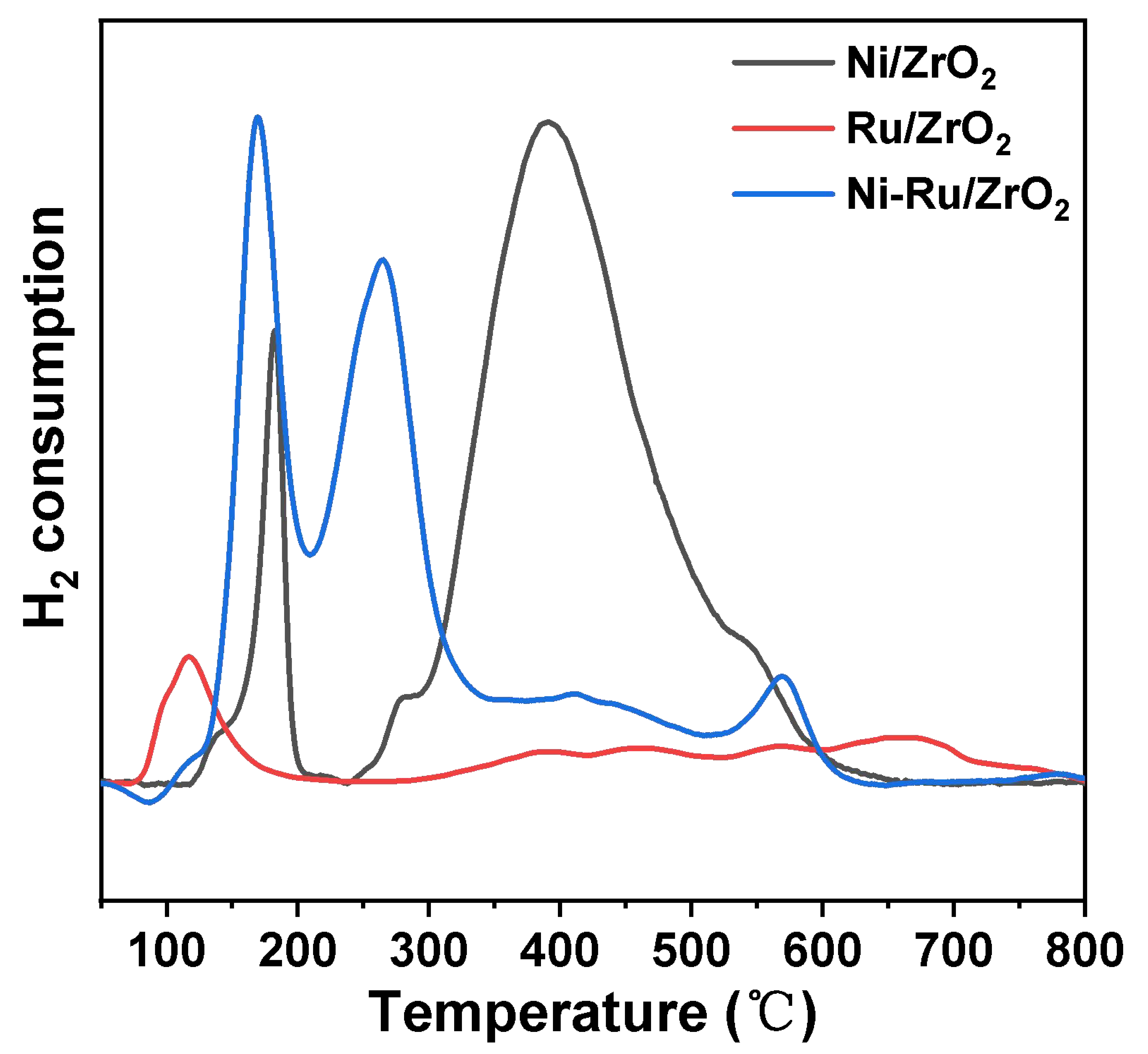


Fig S2 shows the H_2_-TPR images of Ni/ZrO_2_, Ru/ZrO_2_ and Ni-Ru/ZrO_2_. The peak out of Ru/ZrO_2_ prepared by the impregnation method shows the reduction peak of RuO_2_ nanoparticles at 117 °C. The sharp peak at ~180 °C and broad peaks from 240 to 650 °C in Ni/ZrO_2_ correspond to the reduction of small-size and large-size NiO, respectively.

Fig S3. TEM images (a-c) of Ni-Ru/ZrO_2_, HAADF-STEM images and EDX mapping of the corresponding Ni, Ru, Na and Zr (d-g).


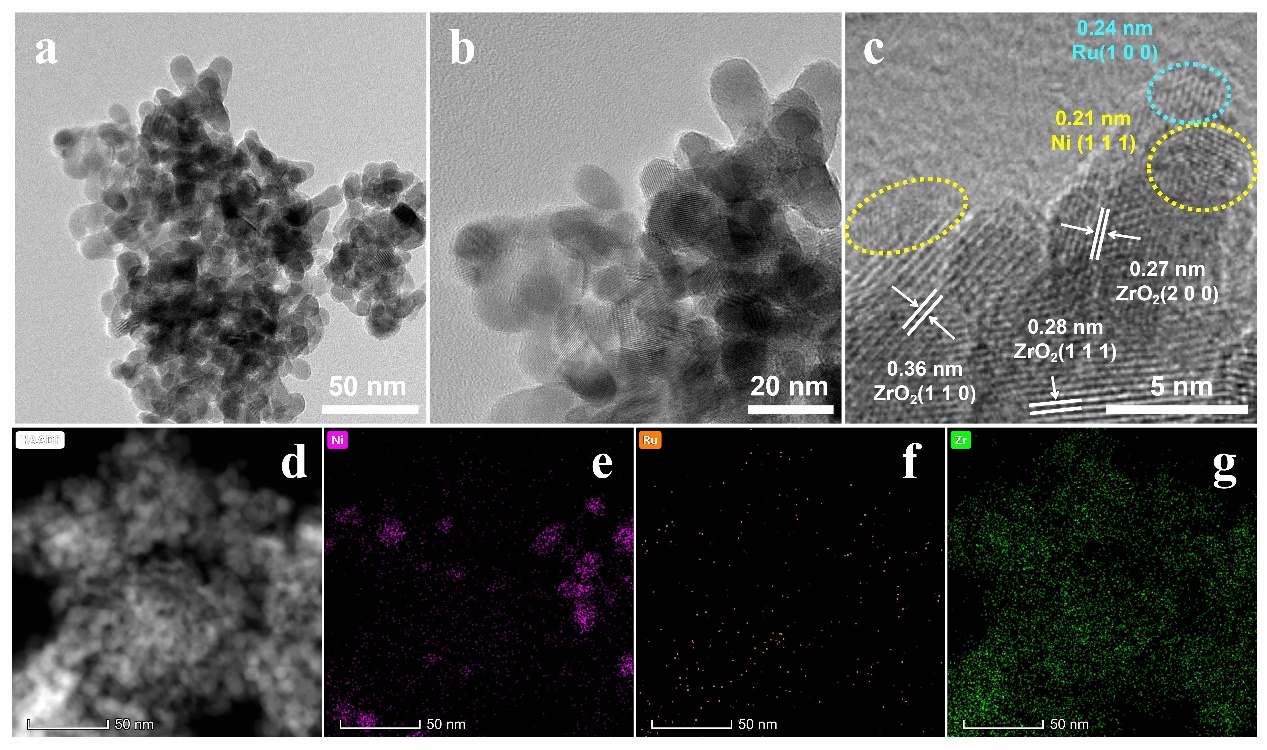


Fig S4. HAADF-STEM image of 0.2Na-Ni-Ru/Ru/ZrO_2_-NW and corresponding EDX mapping of Ni, Ru, Na and Zr.


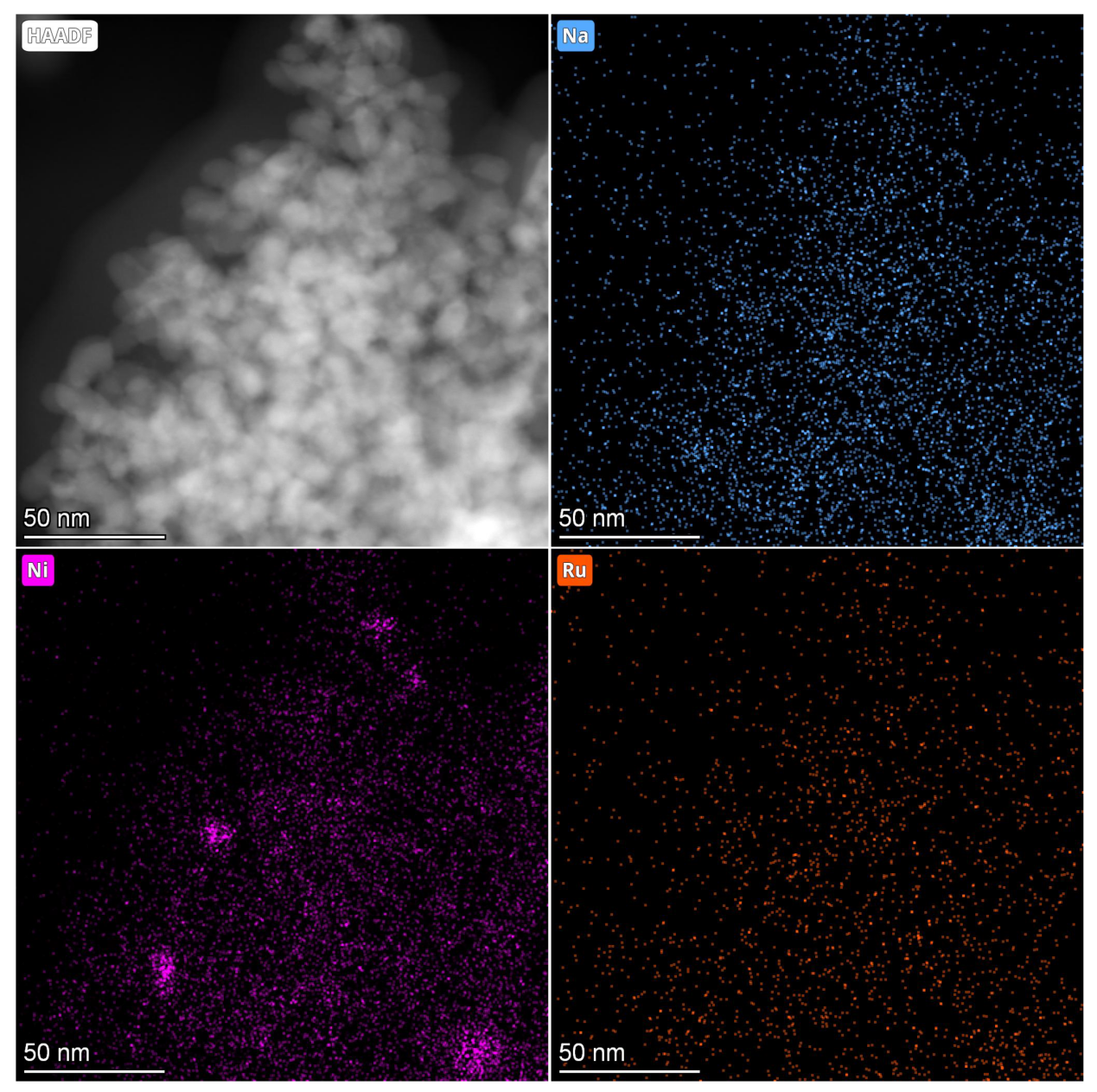


Fig S5. XPS spectra of 0.2Na-Ni-Ru/ZrO_2_-NW and 0.2Na-Ni-Ru/ZrO_2_.

Fig S6. XPS spectra of (a) Na 1s (b) Ru 3p (c) Zr 3d and (d) O 1s before and after the introduction of low Na (0.05), medium Na (0.2), and high Na (0.6) into the photocatalyst Ni-Ru/ZrO_2_.


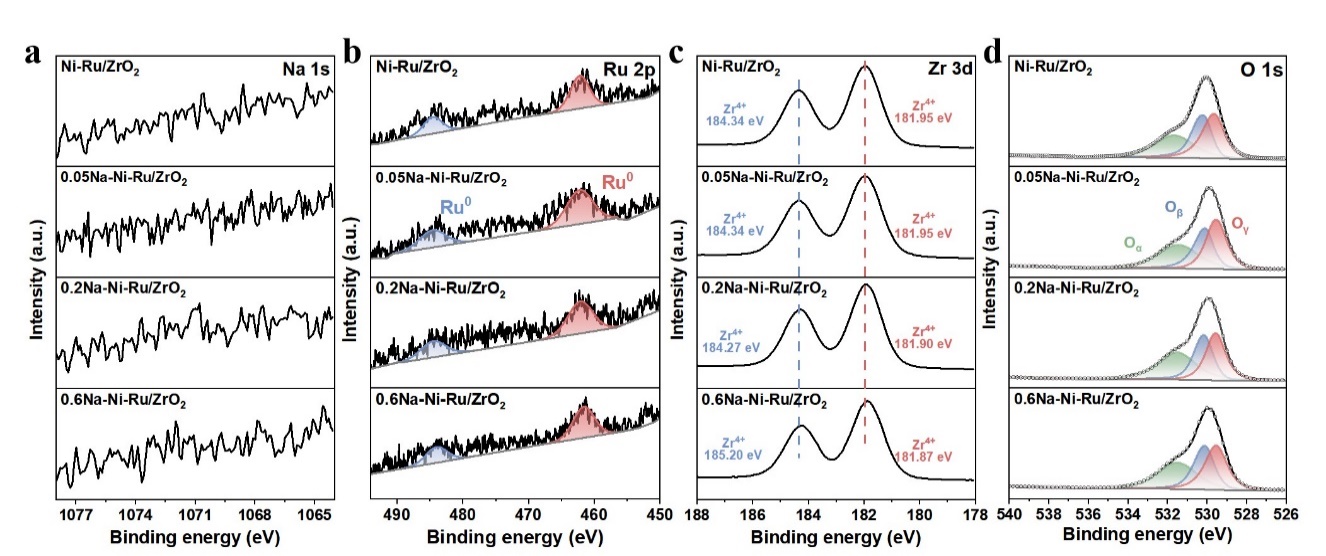


Fig S7. EPR spectra of ZrO_2_, Ni-Ru/ZrO_2_ and 0.2Na-Ni-Ru/ZrO_2_.


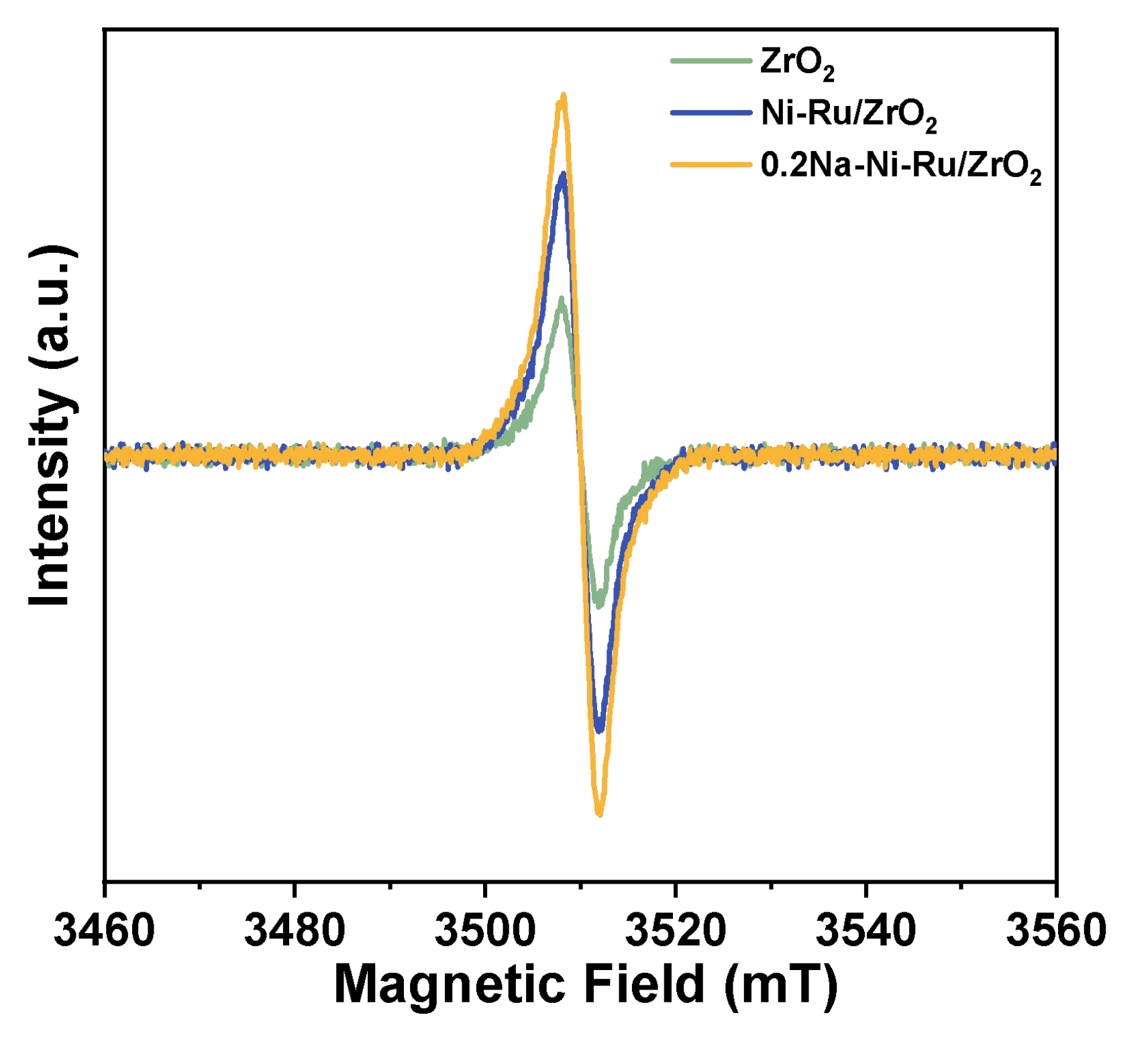


Fig S8. Photocatalytic CO_2_ methanation performance of high and ultra-high Na-introduced xNa-Ni-Ru/ZrO_2_ (x = n_Na_/n_Ni_, 0.4, 0.6, 0.8, and 1.0) series catalysts.


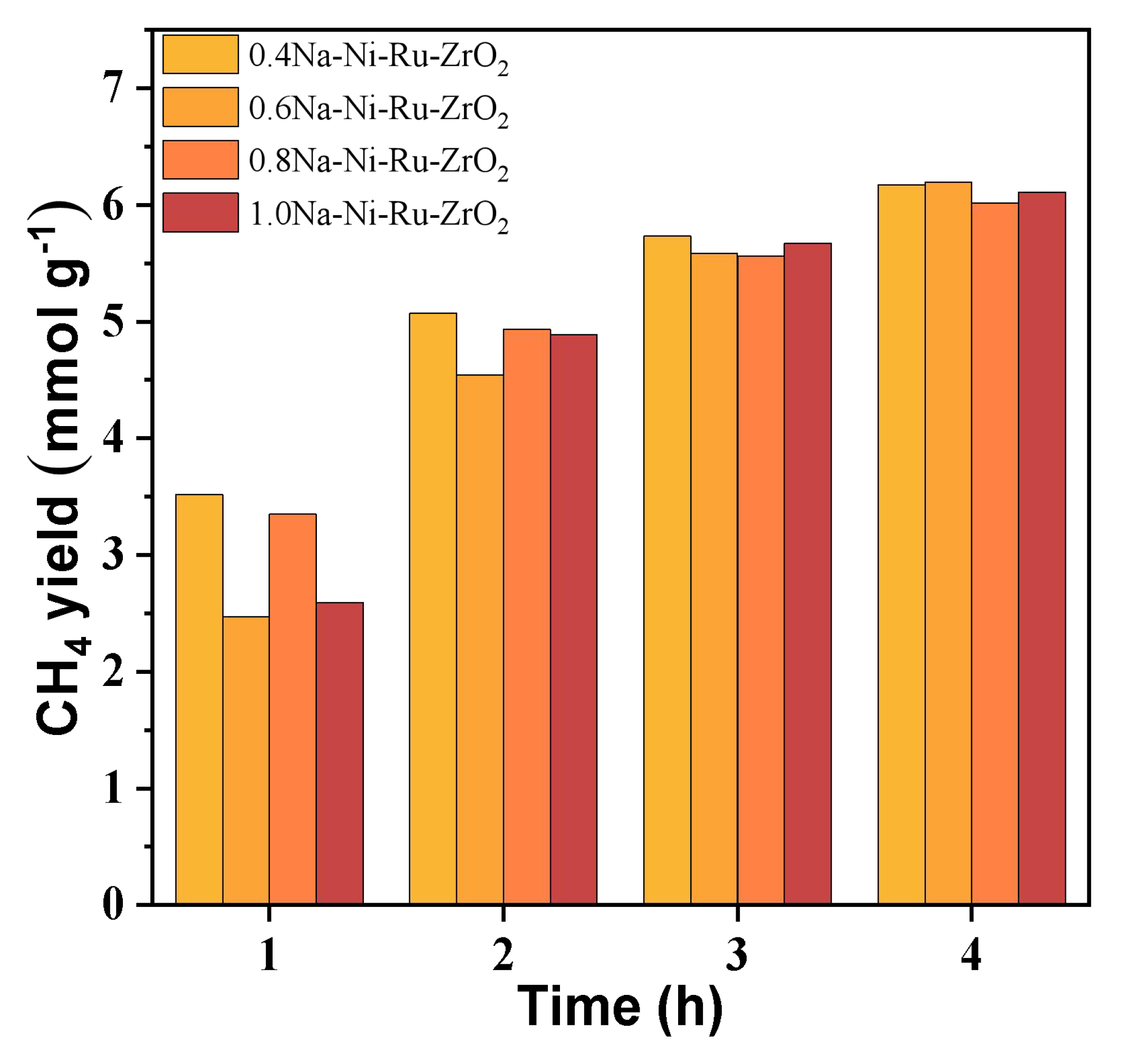


Fig. S9. Characterization comparison of fresh and recycled 0.2Na–Ni–Ru/ZrO_2_ photocatalysts after five reaction cycles. (a) XRD patterns. (b–f) XPS spectra of Zr 3d (b), O 1s (c), Na 1s (d), Ni 2p (e), and Ru 3p (f). (g, h) SEM images before (g) and after (h) reaction cycles. (i) HR-TEM image.


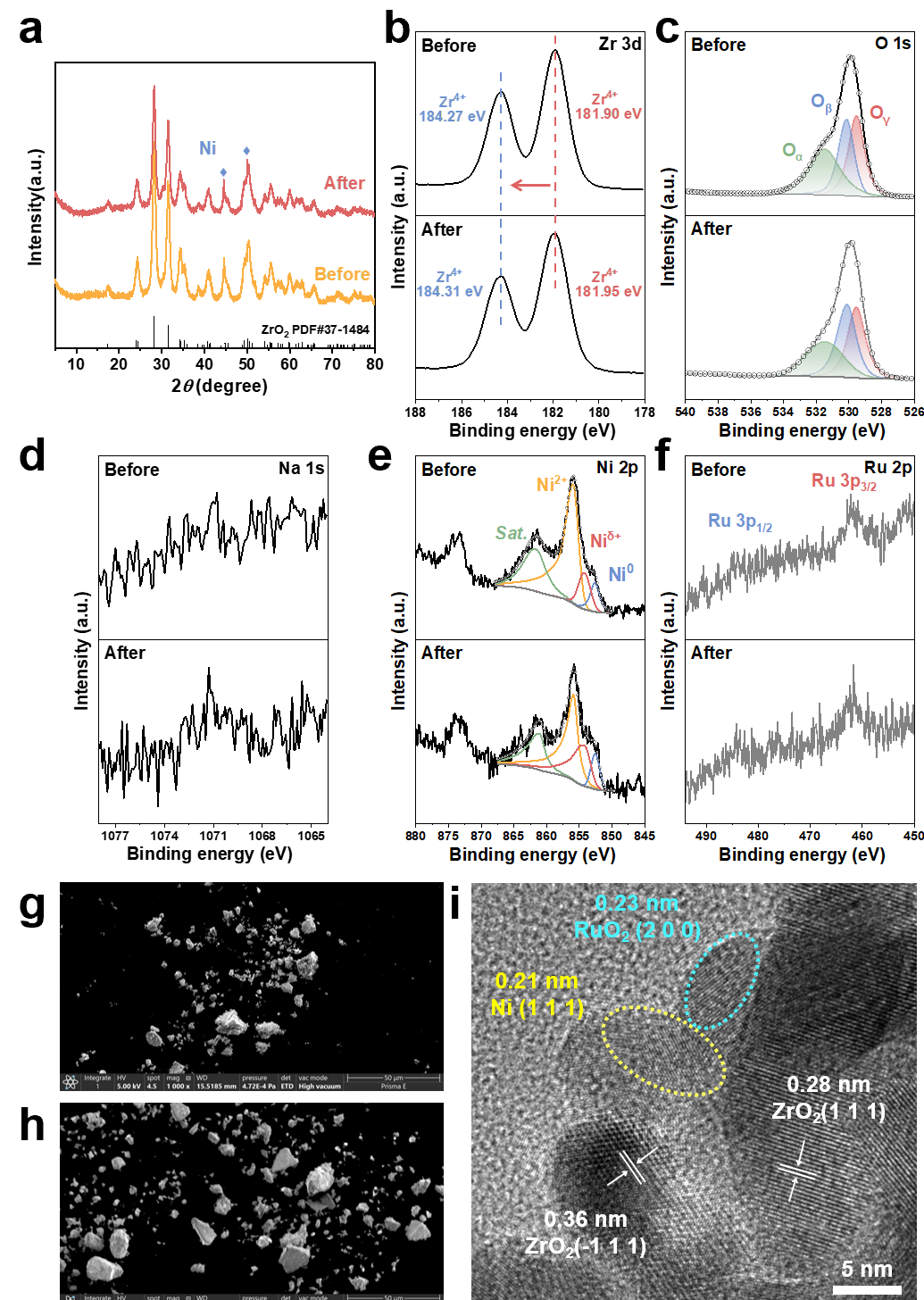


Fig S10. Comparative plots of photocatalytic CO_2_ methanation activities with different alkali-metal promoters (Na *vs.* K).


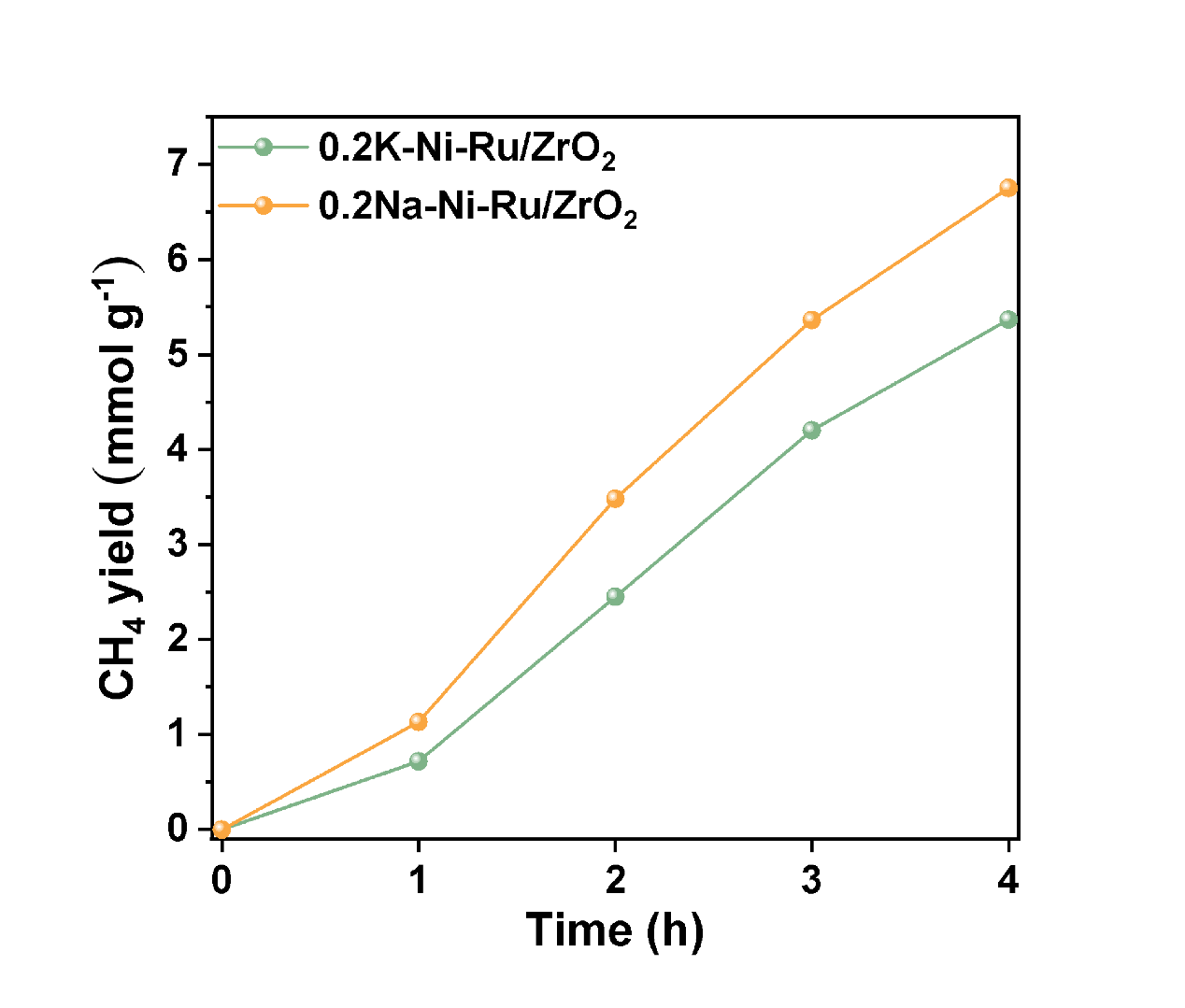


Fig. S11. UV-visible absorption spectra of xNa-Ni-Ru/ZrO_2_ (x = 0.05, 0.1, 0.2, 0.4, 0.6) series photocatalysts.


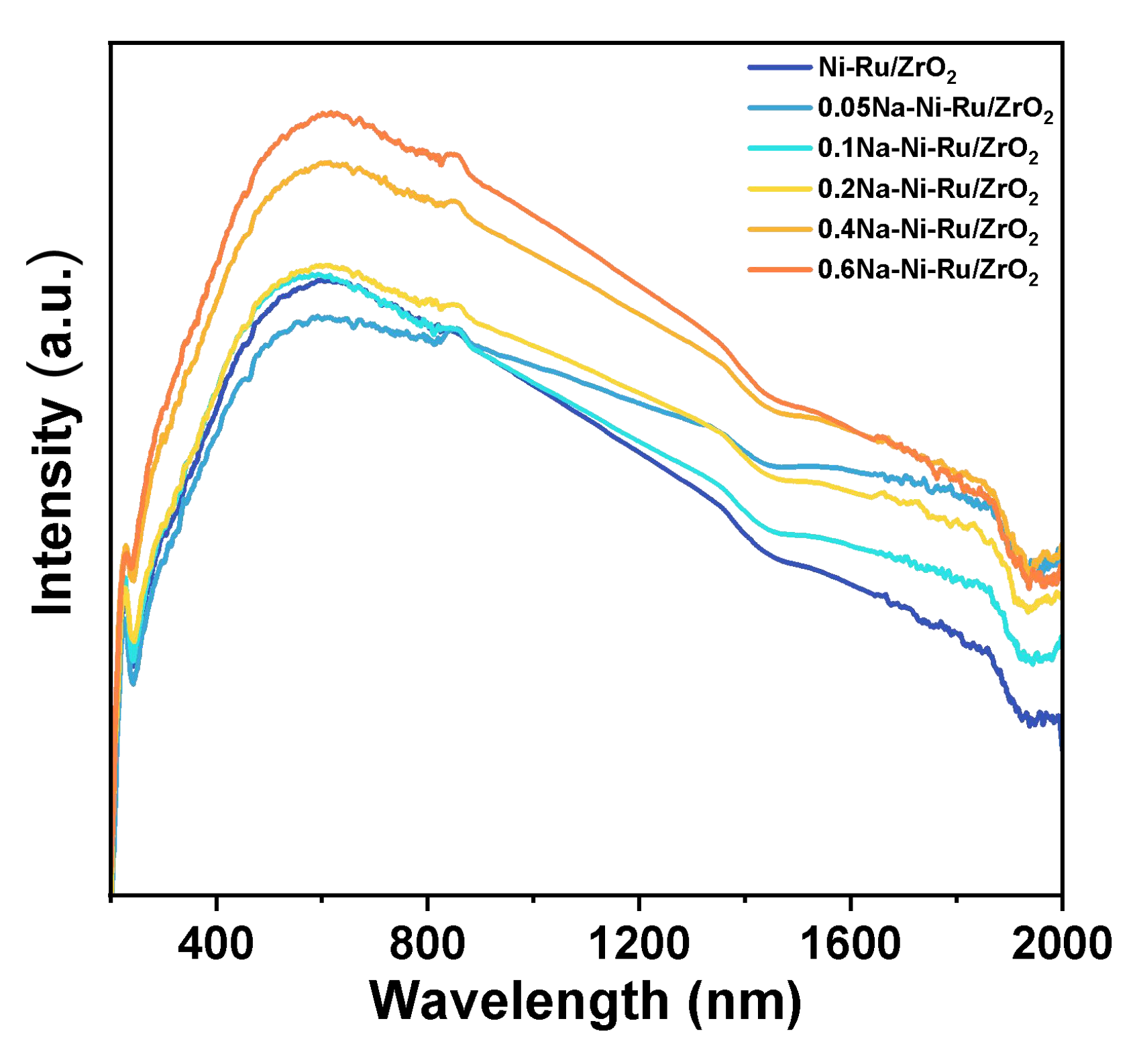


Fig S11 shows the UV-vis absorption spectra of xNa-Ni-Ru/ZrO_2_ (x = 0.05, 0.1, 0.2, 0.4, 0.6) series photocatalysts. The photo absorption ability of the catalysts showed a gradual enhancement with the increase of Na_inter_ content. And the abnormal light absorption performance of the 0.05Na-Ni-Ru/ZrO_2_ sample is related to the larger size of Ni loading.

Fig S12. Infrared thermograms of ZrO_2_, Ni-Ru/ZrO_2_, 0.2Na-Ni-Ru/ZrO_2_, and 0.2Na-Ni-Ru/ZrO_2_-NW after 300 s of light exposure.


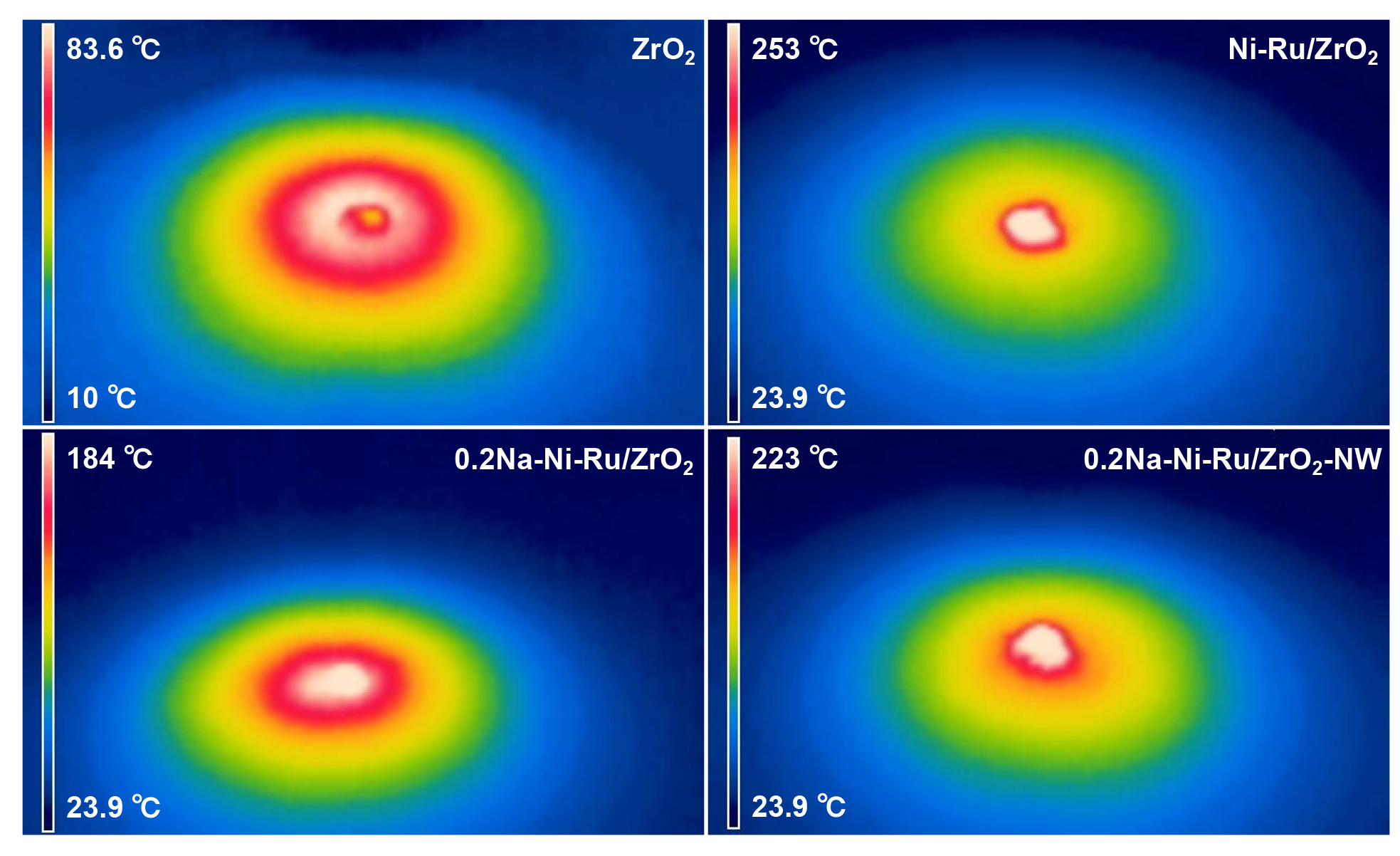


Fig S13. Control experiment results of thermal catalysis conducted at 184 °C in the dark.


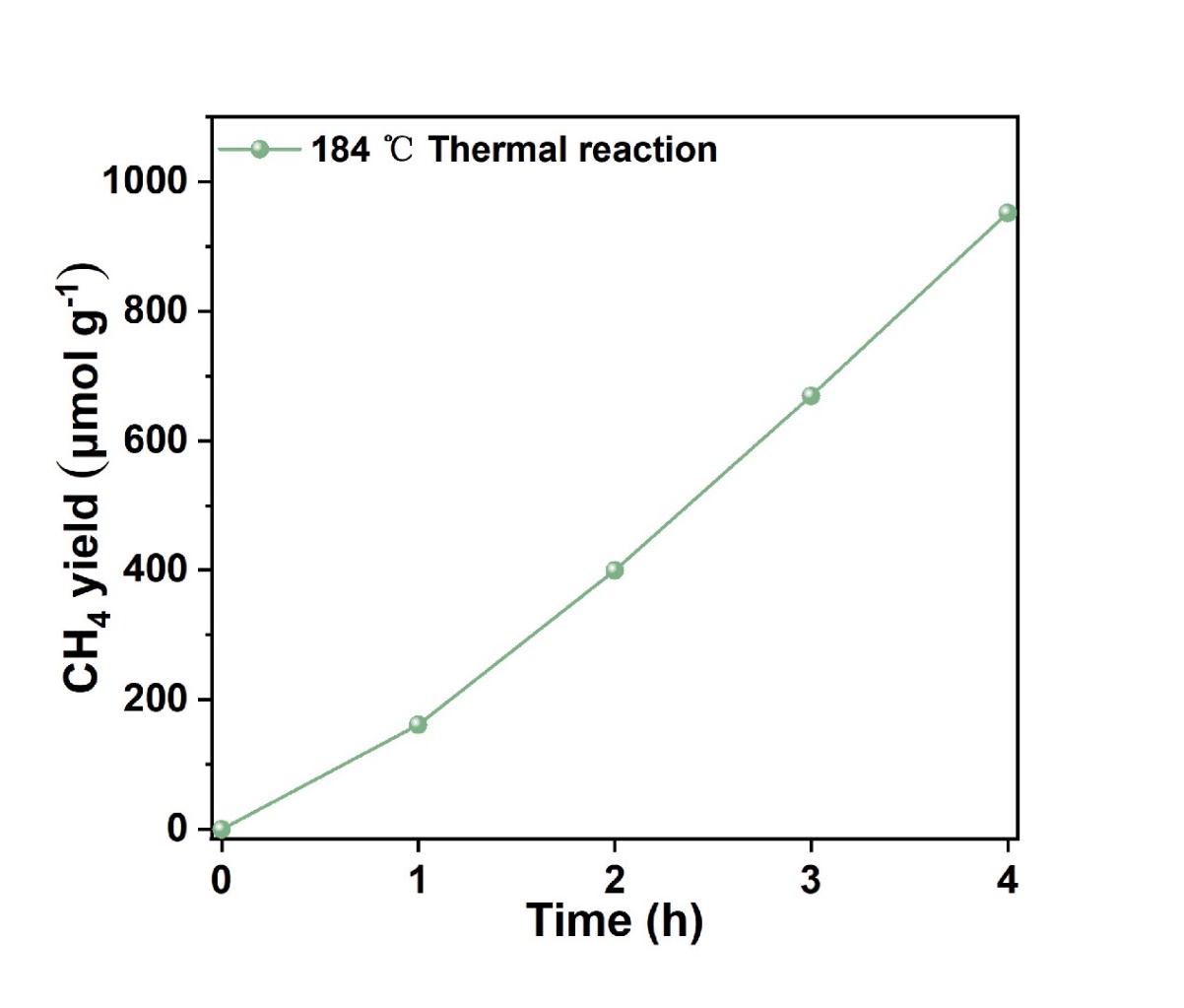


Fig. S14. Comparison of photocatalytic performance of 0.2Na–Ni–Ru/ZrO_2_ under different illumination conditions. (a) Time-dependent CH_4_ yields at different light intensities (0.5, 1.0, and 1.5 W cm⁻^1^) over a 4-hour reaction period. (b) Corresponding bar-chart comparison of CH_4_ production rates calculated from panel (a). (c) Relative photocatalytic activity under full-spectrum (1.5 W cm⁻^1^), visible light (400–800 nm, with optical filter), and infrared light (>800 nm, with optical filter). (d) CH_4_ production rates under identical irradiance (0.5 W cm⁻^1^) comparing full-spectrum, visible (400–800 nm), and infrared (>800 nm) illumination conditions.


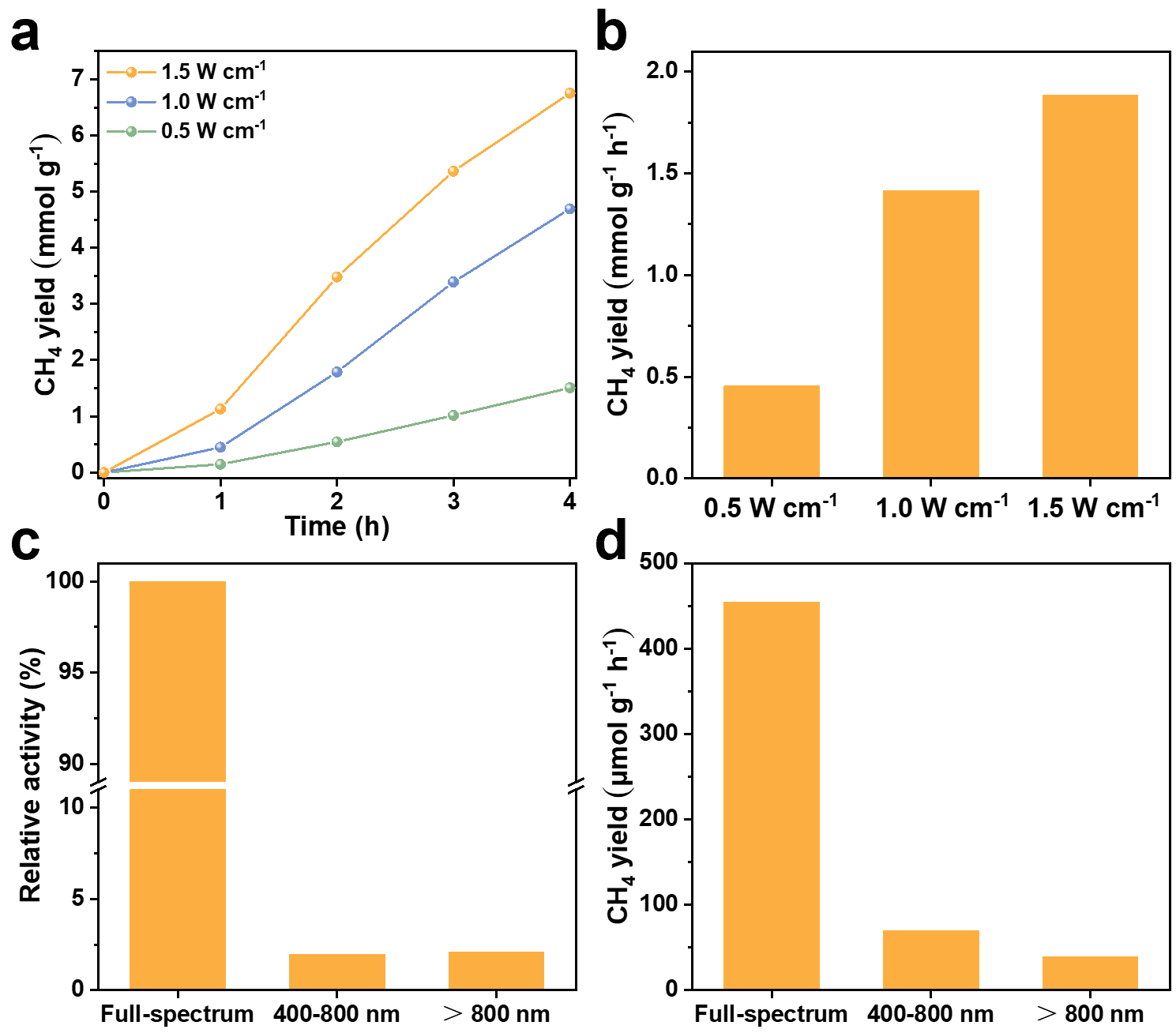


Fig S15. CO_2_-TPD (a) and CH_4_-TPD (b) for 0.2Na-Ni-Ru/ZrO_2_ and 0.2Na-Ni-Ru/ZrO_2_-NW.


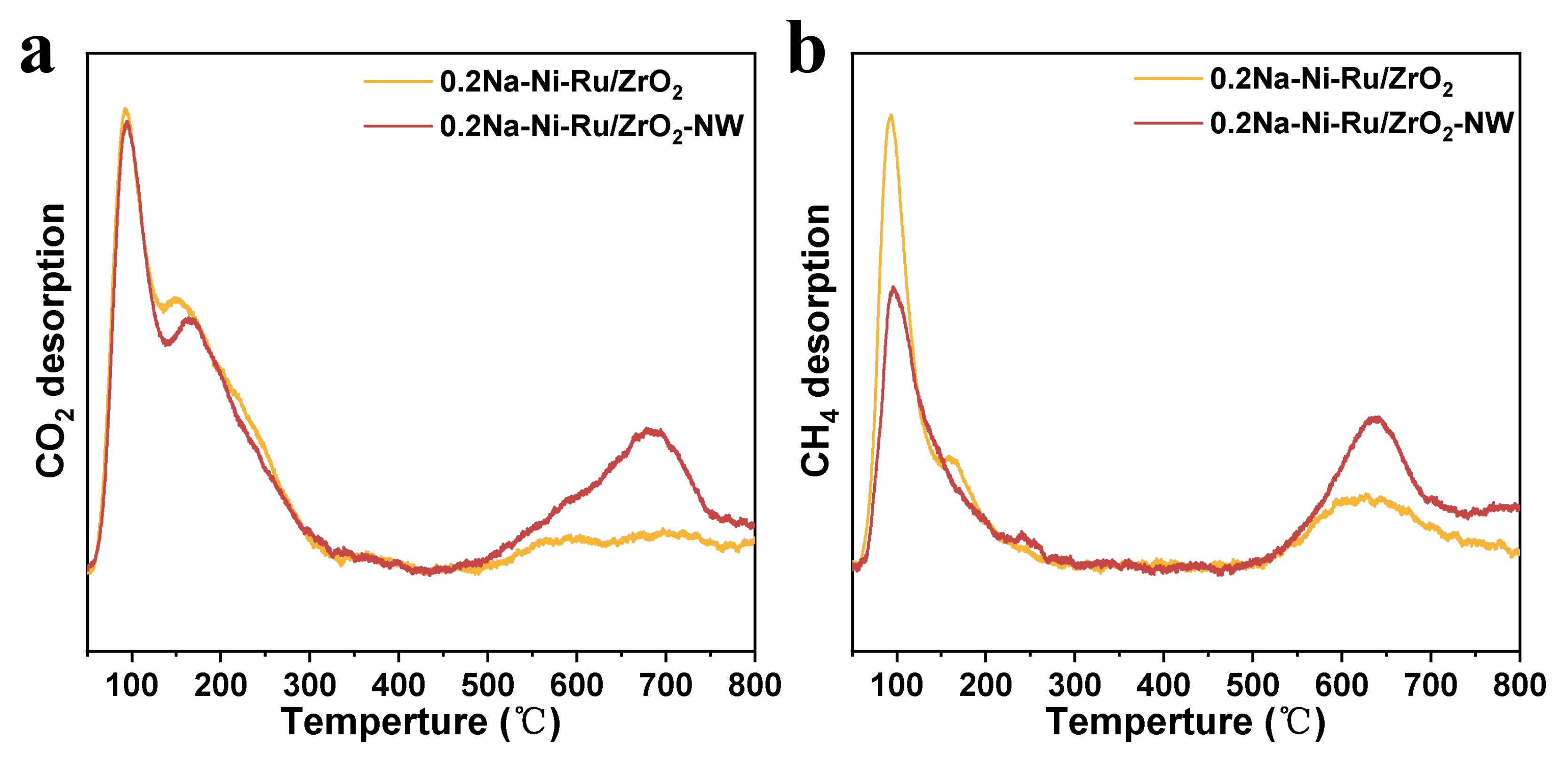


Fig S16. *In-situ* XPS spectra of Ni-Ru/ZrO_2_ in the pristine, H_2_-reduced, light on and light off states for (a) Ru 3p, (b) Zr 3d and (c) O 1s.


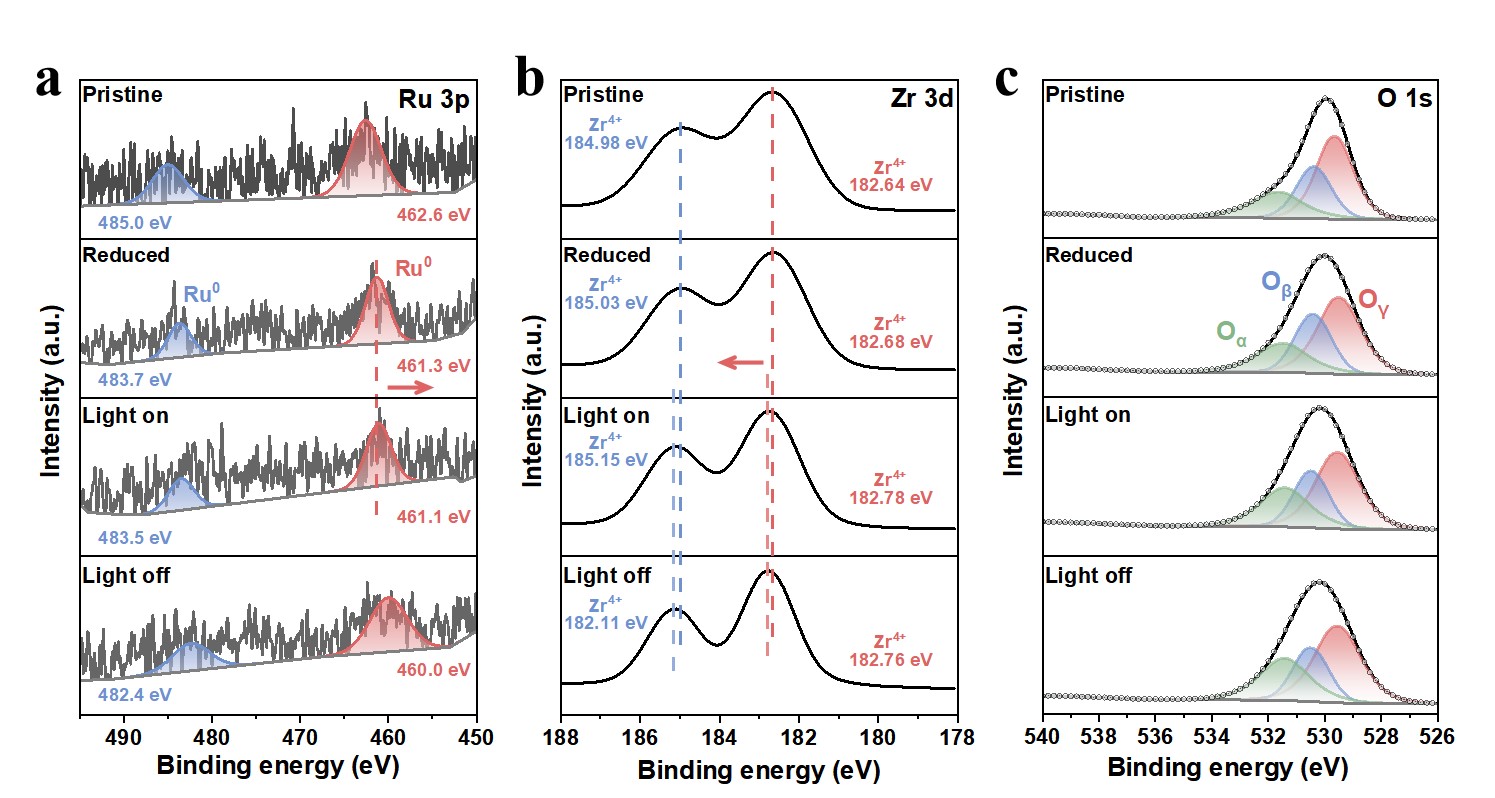


Fig S17. *In-situ* XPS spectra of 0.2Na-Ni-Ru/ZrO_2_ in the pristine, hydrogen-reduced, light on and light off states for (a) Ru 3p, (b) Zr 3d, (c) O 1s and (c) Na 1s.

**
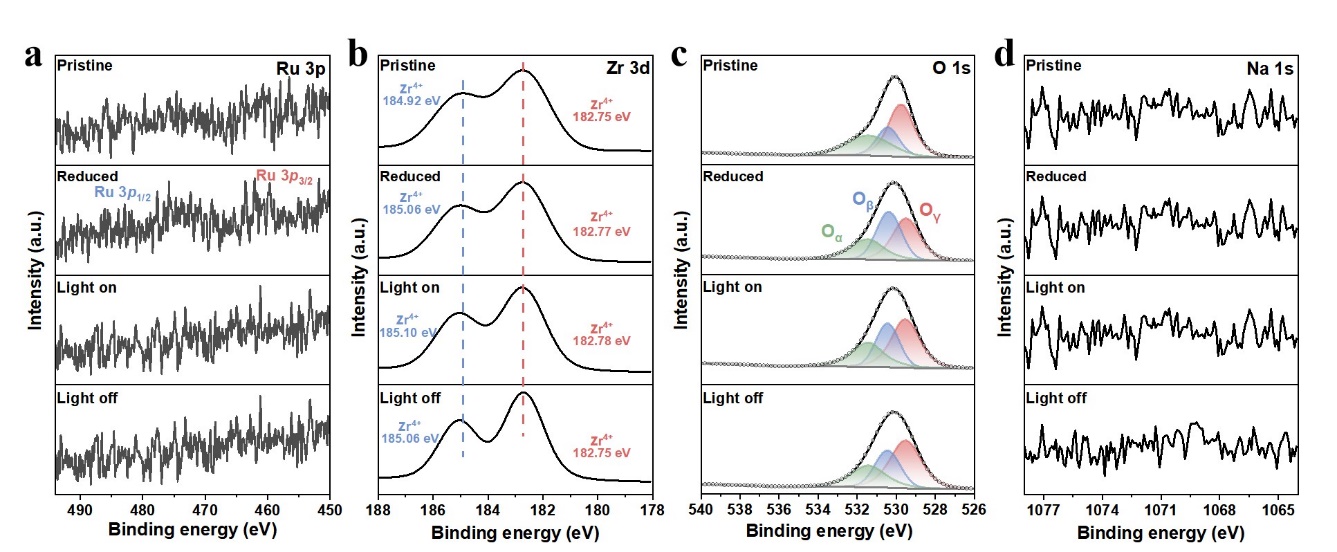
**

Fig S18. *In-situ* DRIFTS of CO_2_ adsorption stage (a) and CO_2_ hydrogenation reaction stage (b) on Ni-RuZrO_2_ photocatalysts. *In-situ* DRIFTS of CO_2_ adsorption stage (c) and CO_2_ hydrogenation reaction stage (d) on 0.2Na-Ni-Ru/ZrO_2_ photocatalysts.


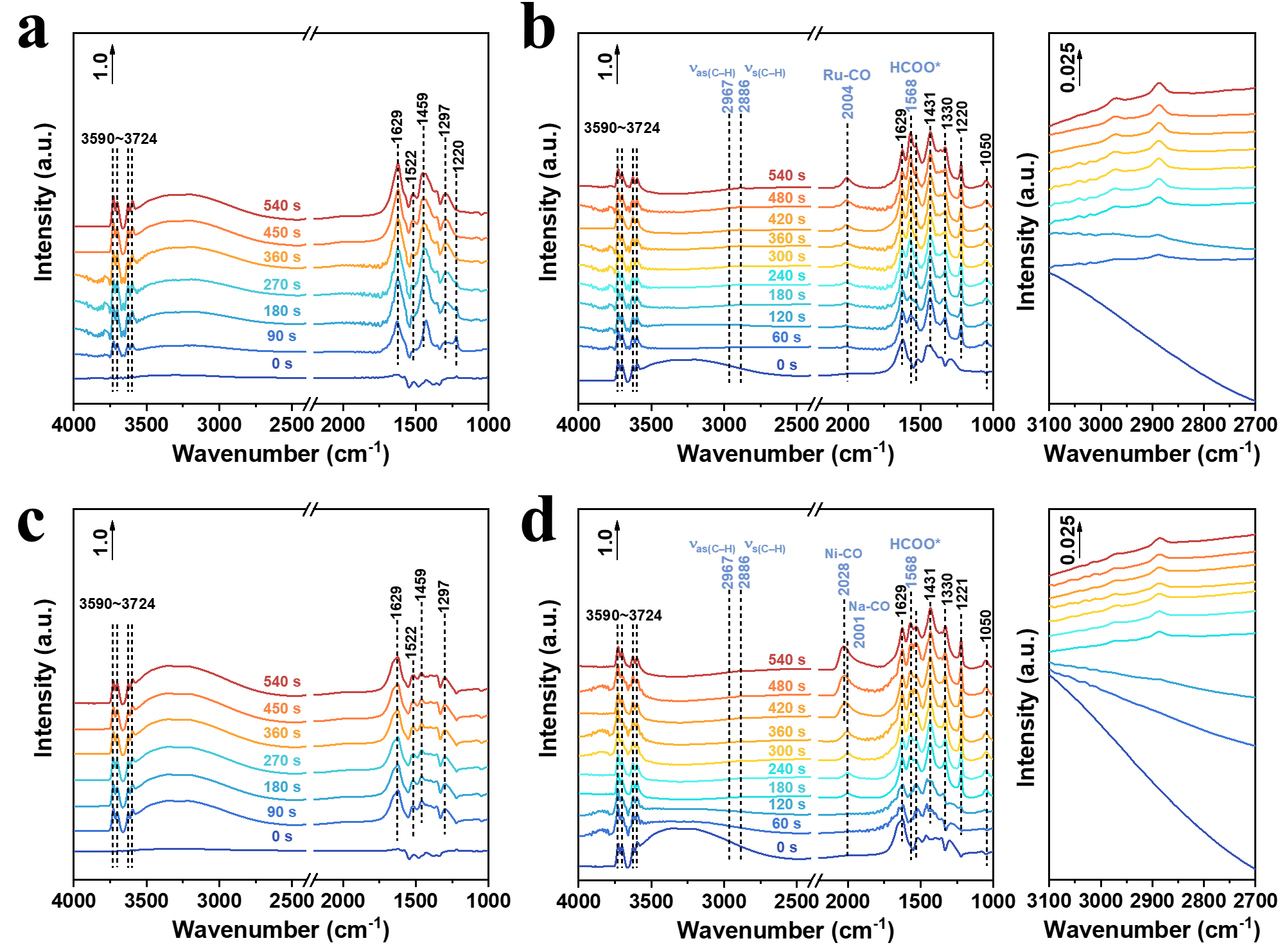


Fig S19. Adsorption energies of CO on Ni-Ru/ZrO_2_ (a) and 0.2Na-Ni-Ru/ZrO_2_ (b).

**
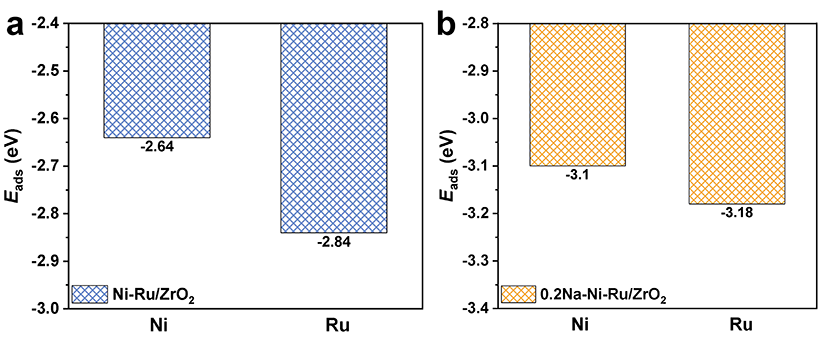
**

Fig S20. The optimized structural models (a-b) in top view and charge density difference (c-d) for Ni-Ru/ZrO_2_ and 0.2Na-Ni-Ru/ZrO_2_. Yellow and cyan regions represent electron density accumulation and depletion, respectively.

**
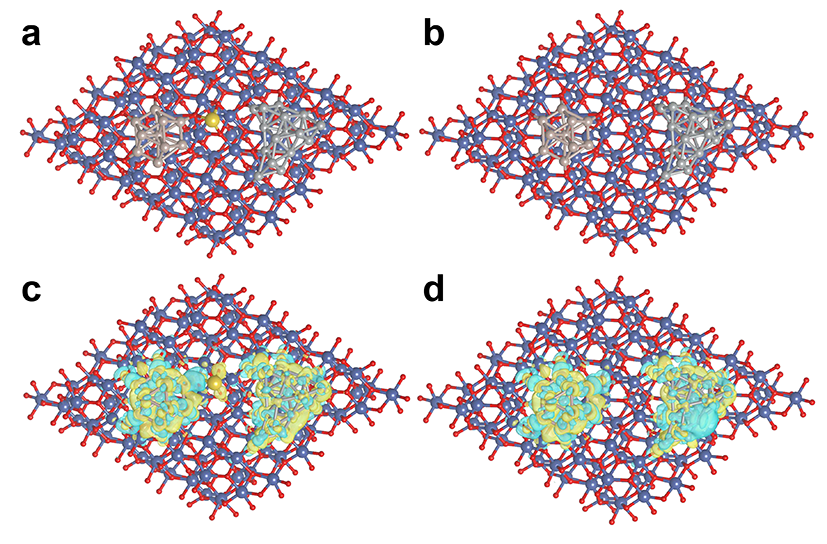
**

Fig S21. Front and side views of the optimized structural models for H_2_ adsorption (a-b) and 2*H adsorption (c-d) on Ni-Ru/ZrO_2_.

**
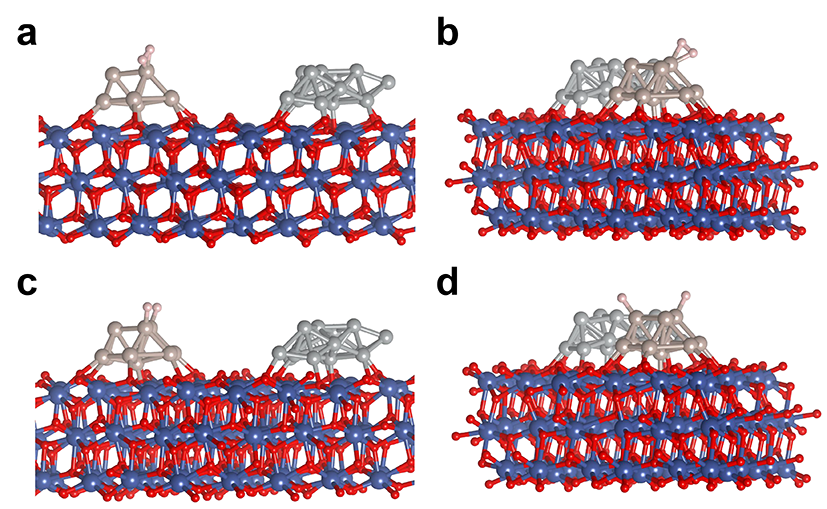
**

Fig S22. Front and side views of the optimized structural models for H_2_ adsorption (a-b) and 2*H adsorption (c-d) on 0.2Na-Ni-Ru/ZrO_2_.

**
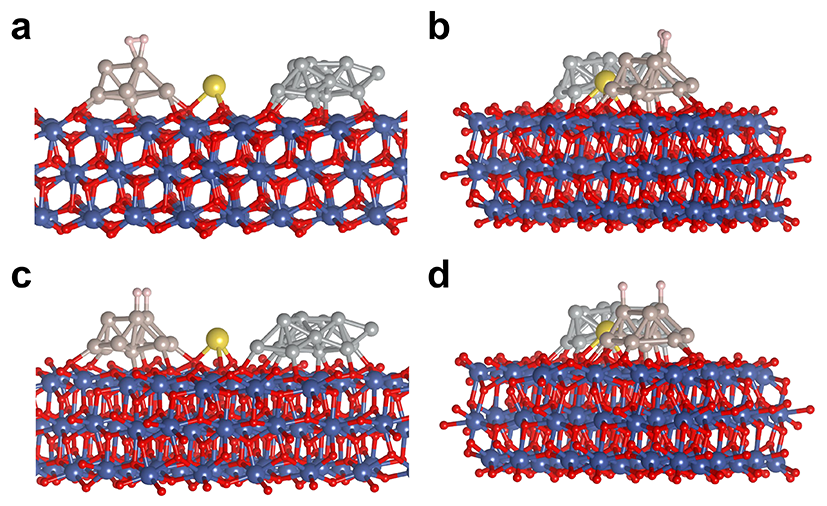
**

Figure S23. Front and side views of the optimized structural models for CO_2_ adsorption (a-b), HCOO* (c-d), and *CO (e-f) on Ni-Ru/ZrO_2_.

**
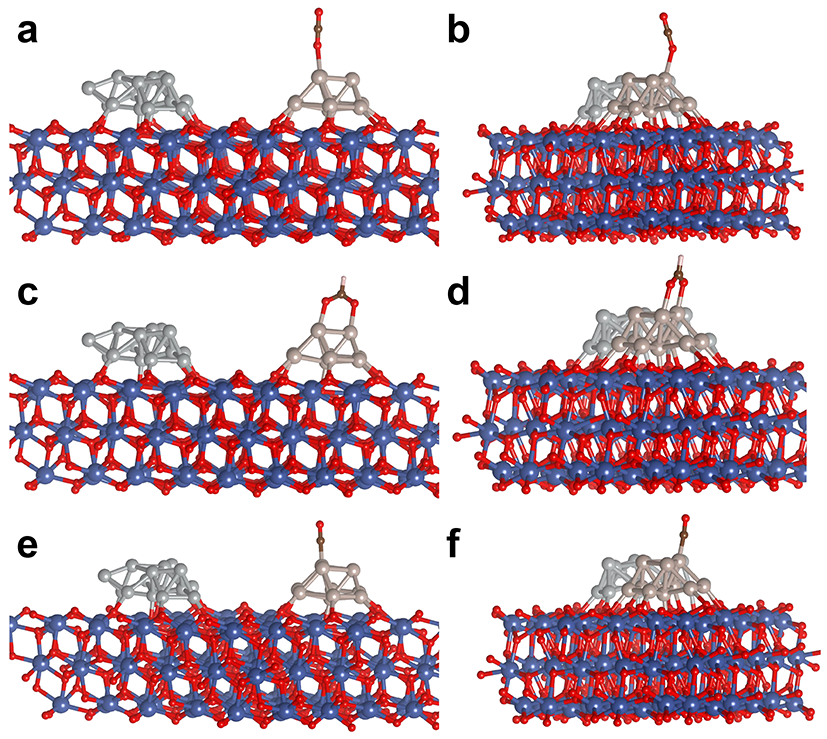
**

Figure S24. Front and side views of the optimized structural models for CO_2_ adsorption (a-b), HCOO* (c-d), and *CO (e-f) on 0.2Na-Ni-Ru/ZrO_2_.

**
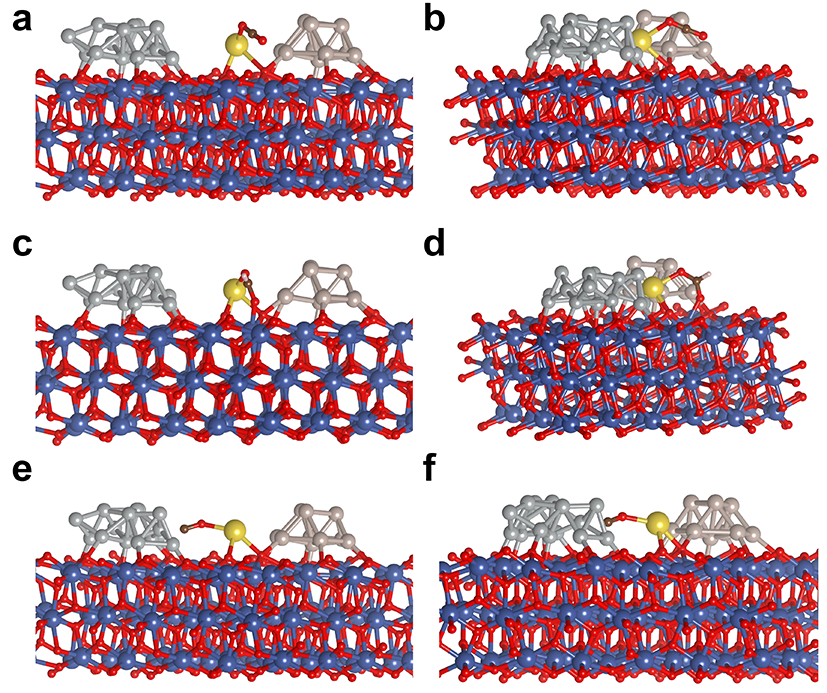
**

| Table S1. The content of Ni, Ru and Na were determined by ICP-OES. | | | | | | |
| --- | --- | --- | --- | --- | --- | --- |
| Sample | Ni-Ru/ZrO_2_ | 0.05Na- Ni-Ru/ZrO_2_ | 0.2Na- Ni-Ru/ZrO_2_ | 0.6Na- Ni-Ru/ZrO_2_ | 1Na- Ni-Ru/ZrO_2_ | 0.2Na- Ni-Ru/ZrO_2_-NW |
| Ni(wt%) | 8.71 | 9.93 | 8.83 | 10.4 | 10 | 8.98 |
| Ru(wt%) | 1.02 | 0.981 | 0.696 | 0.941 | 0.97 | 1.04 |
| Na(wt%) | - | 0.008  (± 2.71E-07) | 0.045  (± 3.21E-06) | 0.039  (± 8.2E-07) | 0.033  (± 1.43E-06) | 0.795  (± 1.18E-06) |

Table S2. Surface analysis of xNa-Ni-Ru/ZrO2 catalysts Ni 2p based on XPS.

| Sample | Ni 2p3/2 BE(eV) | | | |
| --- | --- | --- | --- | --- |
|  |  |  |  |  |
|  | Ni0 | Niδ+ | Ni2+ | *Sat.* |
| Ni-Ru/ZrO_2_ | 852.7 | - | 856.0 | 861.6 |
| 0.05Na-Ni-Ru/ZrO_2_ | 852.7 | 854.3 | 855.8 | 861.3 |
| 0.2Na-Ni-Ru/ZrO_2_ | 852.6 | 854.2 | 856.0 | 861.6 |
| 0.6Na-Ni-Ru/ZrO_2_ | 852.7 | 854.2 | 855.9 | 861.7 |

Table S3. Surface analysis of xNa-Ni-Ru/ZrO_2_ catalysts O 1s based on XPS.

| Sample | O_α_  BE(eV) | O_β_   BE(eV) | O_γ_  BE(eV) | O_α_ (%) | O_β_  (%) | O_γ_ (%) | (O**_α_**+O_β_)/(O_total_) (%) |
| --- | --- | --- | --- | --- | --- | --- | --- |
|  |  |  |  |  |  |  |  |
| Ni-Ru/ZrO_2_ | 531.6 | 530.2 | 529.6 | 31.2 | 31.5 | 37.3 | 62.7 |
| 0.05Na-Ni-Ru/ZrO_2_ | 531.4 | 530.1 | 529.5 | 33.6 | 29.5 | 36.9 | 63.1 |
| 0.2Na-Ni-Ru/ZrO_2_ | 531.5 | 530.2 | 529.5 | 36.1 | 30.4 | 33.5 | 66.5 |
| 0.6Na-Ni-Ru/ZrO_2_ | 531.5 | 530.1 | 529.5 | 34.5 | 30.8 | 34.7 | 65.3 |

Table S4. Surface analysis of Ni-Ru/ZrO_2_ catalysts Ni 2p based on *in-situ* XPS under different conditions.

| Sample | Ni 2p_3/2_ BE(eV) | | |
| --- | --- | --- | --- |
|  |  |  |  |
|  | Ni^0^ | Ni^2+^ | *Sat.* |
| Pristine | 852.7 | 855.5 | 861.2 |
| Reduced | 852.5 | 855.2 | 861.1 |
| Light on | 852.6 | 855.5 | 860.9 |
| Light off | 852.4 | 855.3 | 861.5 |

Table S5. Surface analysis of Ni-Ru/ZrO_2_ catalysts O 1s based on in-situ XPS under different conditions.

| Sample | O_α_  BE(eV) | O_β_   BE(eV) | O_γ_  BE(eV) | O_α_ (%) | O_β_  (%) | O_γ_ (%) | (O**_α_**+O_β_)/(O_total_) (%) |
| --- | --- | --- | --- | --- | --- | --- | --- |
|  |  |  |  |  |  |  |  |
| Pristine | 531.6 | 530.4 | 529.6 | 22.7 | 28.2 | 49.1 | 50.9 |
| Reduced | 531.4 | 530.4 | 529.5 | 22.3 | 29.7 | 48.0 | 52.0 |
| Light on | 531.4 | 530.5 | 529.5 | 27.1 | 26.1 | 46.8 | 53.2 |
| Light off | 531.4 | 530.5 | 529.5 | 28.2 | 24.2 | 47.5 | 52.4 |

Table S6. Surface analysis of 0.2Na-Ni-Ru/ZrO_2_ catalysts Ni 2p based on in-situ XPS under different conditions.

| Sample | Ni 2p_3/2_ BE(eV) | | | |
| --- | --- | --- | --- | --- |
|  |  |  |  |  |
|  | Ni^0^ | Ni^δ+^ | Ni^2+^ | *Sat.* |
| Pristine | 852.6 | 854.2 | 855.6 | 861.6 |
| Reduced | 852.6 | 854.2 | 855.6 | 862.2 |
| Light on | 852.6 | 854.2 | 855.6 | 861.5 |
| Light off | 852.4 | 854.4 | 855.6 | 860.5 |

Table S7. Surface analysis of 0.2Na-Ni-Ru/ZrO_2_ catalysts O 1s based on in-situ XPS under different conditions.

| Sample | O_α_  BE(eV) | O_β_   BE(eV) | O_γ_  BE(eV) | O_α_ (%) | O_β_  (%) | O_γ_ (%) | (O**_α_**+O_β_)/(O_total_) (%) |
| --- | --- | --- | --- | --- | --- | --- | --- |
|  |  |  |  |  |  |  |  |
| Pristine | 531.4 | 530.4 | 529.7 | 30.3 | 22.2 | 47.5 | 52.5 |
| Reduced | 531.4 | 530.4 | 529.5 | 23.4 | 38.4 | 38.2 | 61.8 |
| Light on | 531.4 | 530.4 | 529.5 | 25.8 | 30.7 | 43.5 | 56.5 |
| Light off | 531.4 | 530.4 | 529.5 | 24.4 | 28.6 | 47.0 | 53.0 |

Table S8. EXAFS fitting parameters at the Ni K-edge for Ni-Ru/ZrO_2_ and 0.2Na-Ni-Ru/ZrO_2_.

| Sample | Shell | R(Å)^a^ | C.N.^b^ | *σ^2^*(Å)^c^ | ΔE^0^(eV)^d^ | *R* factor(%)^e^ |
| --- | --- | --- | --- | --- | --- | --- |
| Ni-Ru/ZrO_2_ | Ni-Ni | 2.50 | 6.29 | 0.006 | 0.003 | 0.014 |
| 0.2Na-Ni-Ru/ZrO_2_ | Ni-Ni | 2.47 | 7.07 | 0.005 | -0.017 | 0.012 |

^a^ *R*: Average distance between absorber and backscattered atoms.

^b^ Coordination number, determined by curve fitting.

^c^ Debye-Waller factor.

^d^ Inner potential correction.

^e^ *R* factor: goodness of fit, If R factor <5%, consistent with broadly correct models.

Table S9. Comparison of CH_4_ yield of our work with related reports on photocatalyst CO_2_ methanation.

| Catalysts | Light source | Temperature(℃) | CH_4_ rate | Ref. |
| --- | --- | --- | --- | --- |
| 0.2Na-Ni-Ru/ZrO_2_ | 300 W Xe lamp 1.5 W cm^-2^ |  | 1882.7 μmol g_cat_^-1^ h^-1^ | This work |
|  |  |  |  |  |
| Ni-ZrO_2_ | 500 W Xe arc lamp 142 mW cm^−2^ |  | 0.98 mmol g_cat_^-1^ h^-1^ | ^[7]^ |
|  |  |  |  |  |
| In_2_O_3_@Ni | 300 W Xe lamp |  | 1043 μmol g^−1^ h^−1^ | ^[8]^ |
|  |  |  |  |  |
| Co-ss@SiO_2_ | 300 W Xe lamp 2.5 W cm^-2^ |  | 2.3 mmol g_Co_^-1^ h^-1^ | ^[9]^ |
|  |  |  |  |  |
| Ni/BN | 300 W Xe lamp |  | 2.03 mol g_Ni_^−1^ h^−1^ | ^[10]^ |
|  |  |  |  |  |
| 0.35%Ru@Ni_2_V_2_O_7_ | 300 W Xe lamp 2.0 W cm^-2^ |  | 114.9 mmol g_cat_^-1^ h^-1^ | ^[11]^ |
|  |  |  |  |  |
| Ru/Al_2_O_3_ | 300 W Xe lamp |  | 18.16 mol g_Ru_^-1^ h^-1^ | ^[12]^ |
| Ni/Al_2_O_3_ |  |  | 2.3 mol g_Ni_^-1^ h^-1^ |  |
| Ru_0.76_Ni_0.24_/TiO_2_ | Xe lamp 1.8 W cm^-2^ | 250 | 3.58 mmol g_metal_^-1^ h^-1^ | ^[13]^ |
|  |  |  |  |  |
| Ru/MnCo_2_O_4_ | 300 W Xe lamp 1.25 W cm^-2^ | 230 | 66.3 mmol g_cat_^-1^ h^-1^ | ^[14]^ |
|  |  |  |  |  |
| Ru-Al_2_O_3_-x-L | 300 W Xe lamp 2.0 W cm^-2^ | 240 | 12.35 mmol g_Ru_^-1^ h^-1^ | ^[15]^ |
|  |  |  |  |  |

**Reference**

[1] G. Kresse, J. Furthmüller, Efficiency of ab-initio total energy calculations for metals and semiconductors using a plane-wave basis set, *Comp. Mater. Sci.* **1996**. 615-50.

[2] P.E. Blöchl, Projector augmented-wave method, *Phys. Rev. B.* **1994**, 50 17953-17979.

[3] J.P. Perdew, K. Burke, M. Ernzerhof, Generalized gradient approximation made simple, *Phy. Rev. Lett.* **1996**, 77, 3865-3868.

[4] Li, S.; Xu, Y.; Wang, H.; Teng, B.; Liu, Q.; Li, Q.; Xu, L.; Liu, X.; Lu, J. Tuning the CO_2_ Hydrogenation Selectivity of Rhodium Single‐Atom Catalysts on Zirconium Dioxide with Alkali Ions. *Angew. Chem. Int. Ed.* **2023,** *62,* e202218167.

[5] F. Denis Romero, S. J. Burr, J. E. McGrady, D. Gianolio, G. Cibin and M. A. Hayward, SrFe_0.5_Ru_0.5_O_2_: Square-Planar Ru^2+^ in an Extended Oxide, *J. Am. Chem. Soc.* **2013**, 135, 1838-1844.

[6]H. Wang, C. Zhen, D. Xu, X. Wu, L. Ma, D. Zhao and D. Hou, Multiple magnetic phase transitions in Ni_x_Mn_1-x_Co_2_O_4_, *Ceram. Int.* **2020**, 46, 16126-16134.

[7] H. Zhang, T. Itoi, T. Konishi and Y. Izumi, Efficient and Selective Interplay Revealed: CO_2_ Reduction to CO over ZrO_2_ by Light with Further Reduction to Methane over Ni^0^ by Heat Converted from Light, *Angew. Chem. Int. Ed.* **2021**, 60, 9045-9054.

[8] F. Raziq, C. Feng, M. Hu, S. Zuo, M. Z. Rahman, Y. Yan, Q.-H. Li, J. Gascon, and H. Zhang, Isolated Ni Atoms Enable Near-Unity CH_4_ Selectivity for Photothermal CO_2_ Hydrogenation, *J. Am. Chem. Soc.* **2024**, 146, 21008−21016.

[9] M. Cai, C. Li, X. An, B. Zhong, Y. Zhou, K. Feng, S. Wang, C. Zhang, M. Xiao, Z. Wu, J. He, C. Wu, J. Shen, Z. Zhu, K. Feng, J. Zhong, and L. He, Supra-Photothermal CO_2_ Methanation over Greenhouse-Like Plasmonic Superstructures of Ultrasmall Cobalt Nanoparticles, *Adv. Mater.* **2024**, 36, 2308859.

[10] X. Zhu, H. Zong, C. J. Vi. Pérez, H. Miao, W. Sun, Z. Yuan, S. Wang, G. Zeng, H. Xu, Z. Jiang and G. A. Ozin, Supercharged CO2 Photothermal Catalytic Methanation: High Conversion, Rate, and Selectivity, *Angew. Chem. Int. Ed.* **2023**, 62, e202218694.

[11] Y. Chen, Y. Zhang, G. Fan, L. Song, G. Jia, H. Huang, S. Ouyang, J. Ye, Z. Li and Z. Zou, Cooperative catalysis coupling photo-/photothermal effect to drive Sabatier reaction with unprecedented conversion and selectivity, *Joule*. **2021**, 5, 3235-3251.

[12] X. Meng, T. Wang, L. Liu, S. Ouyang, P. Li, H. Hu, T. Kako, H. Iwai, A. Tanaka, and J. Ye, Photothermal Conversion of CO_2_ into CH_4_ with H_2_ over Group VIII Nanocatalysts: An Alternative Approach for Solar Fuel Production, *Angew. Chem. Int. Ed.* **2014**, 53, 11478 –11482.

[13] C. Guo, L. Wang, Y. Tang, Z. Yang, Y. Zhao, Y. Jiang, X. Wen and F. Wang, Enhanced Photo-Thermal CO_2_ Methanation with Tunable Ru_x_Ni_1-x_ Catalytic Sites: Alloying Beyond Pure Ru, *Adv. Funct. Mater.* **2024**, 2414931.

[14] C. Guo, Y. Tang, Z. Yang, T. Zhao, J. Liu, Y. Zhao and F. Wang, Reinforcing the Efficiency of Photothermal Catalytic CO_2_ Methanation through Integration of Ru Nanoparticles with Photothermal MnCo_2_O_4_ Nanosheets, *ACS Nano*, **2023**, 17, 23761-23771.

[15] X. Liu, C. Xing, F. Yang, Z. Liu, Y. Wang, T. Dong, L. Zhao, H. Liu and W. Zhou, Strong Interaction over Ru/Defects-Rich Aluminium Oxide Boosts Photothermal CO_2_ Methanation via Microchannel Flow-Type System, *Adv. Energy Mater.* **2022**, 12, 2201009.
